# PPY‐Induced iCAFs Cultivate an Immunosuppressive Microenvironment in Pancreatic Cancer

**DOI:** 10.1002/advs.202413432

**Published:** 2025-03-31

**Authors:** Mengdie Cao, Wang Peng, Bin Cheng, Ronghua Wang, Wei Chen, Luyao Liu, Hai Huang, Shiru Chen, Haochen Cui, JingWen Liang, Qiaodan Zhou, Si Xiong, Shuya Bai, Luoxia Liu, Yuchong Zhao

**Affiliations:** ^1^ Department of Gastroenterology and Hepatology Tongji Hospital Tongji Medical College Huazhong University of Science and Technology Wuhan 430030 China; ^2^ Hubei Key Laboratory of Hepato‐Pancreato‐Biliary Diseases Tongji Hospital Tongji Medical College Huazhong University of Science and Technology Wuhan 430030 China; ^3^ Department of Surgery University of Pittsburgh School of Medicine Pittsburgh PA 15213 USA; ^4^ School of Life Sciences The Chinese University of Hong Kong Shatin, New Territories Hong Kong 999077 China; ^5^ Department of Nuclear Medicine Tongji Hospital Tongji Medical College Huazhong University of Science and Technology Wuhan 430030 China

**Keywords:** cancer immunosuppressive microenvironment, hormonal disorders and cancer, immunotherapy, inflammatory cancer‐associated fibroblast, pancreatic polypeptide, stromal plasticity

## Abstract

Pancreatic ductal adenocarcinoma (PDAC) is characterized by cancer cells surrounded by affluent stromal components, which may underlie their limited response to various therapeutic interventions, including immunotherapy. Inflammatory cancer‐associated fibroblasts (iCAFs), a crucial subset of CAFs within the PDAC microenvironment, play a pivotal role in shaping an immunosuppressive microenvironment. In this study, single‐cell RNA sequencing analysis is performed to screen for cancer cells‐secreted proteins associated with iCAF induction, and PPY (pancreatic polypeptide) is validated as a potent inducer. Unlike previously reported iCAF inducers, PPY is a gastrointestinal hormone predominantly expressed in the pancreas, suggesting that targeting it may have minimal systemic effects. Multiplex immunohistochemistry (mIHC) on human PDAC tissue microarrays, orthotopic allograft mouse models, and co‐culture experiments are utilized to validate the crucial role of PPY in iCAF induction. Mechanistic studies integrating mRNA sequencing, immunoprecipitation‐mass spectrometry, and molecular docking reveal that PPY induces iCAFs by activating the non‐canonical NF‐κB pathway through EGFR. Importantly, targeting PPY enhanced the efficacy of anti‐PD‐1 immunotherapy in KPC (*Kras* ^LSL‐G12D/+^; *Trp53* ^LSL‐R172H/+^; *Pdx1*‐Cre) mice, as evidenced by reduced tumor burden on PET‐CT imaging and improved survival. This research is expected to provide a novel strategy for improving immunotherapy in PDAC by targeting a key inducer of iCAFs.

## Graphical abstract

Pancreatic ductal adenocarcinoma features abundant stromal components that hinder therapy efficacy. Single‐cell RNA sequencing analysis combined with experiments identified PPY, a gastrointestinal hormone, as a novel inducer of immunosuppressive iCAFs. Mechanistic studies revealed that PPY activates the non‐canonical NF‐κB pathway via EGFR. Targeting PPY enhanced anti‐PD‐1 immunotherapy in the KPC mouse model, suggesting a new strategy for improving PDAC immunotherapy.

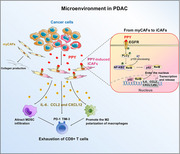

## 1 Introduction

Pancreatic ductal adenocarcinoma (PDAC), a devastating disease with a 5‐year survival rate of ≈10%, exhibits poor responsiveness to various treatment modalities, including immunotherapy.^[^ ^1^, ^2^, ^3^ ^]^ Generally considered as an immunologically “cold” tumor, pancreatic cancer is characterized by a deficient immune cell population within the tumor microenvironment (TME) for effective recognition and elimination of cancer cells, while exhibiting an abundance of immunosuppressive cells, such as myeloid‐derived suppressor cells (MDSCs) and tumor‐associated macrophages (TAMs).^[^ ^4^, ^5^ ^]^ Understanding and modulating this “cold” immunological landscape may be key to improving immunotherapy outcomes in PDAC.^[^ ^3^, ^4^, ^5^, ^6^ ^]^

A hallmark of PDAC is its extensive desmoplastic stroma, which creates a unique tumor microenvironment (TME) that shields cancer cells from effective immune responses.^[^ ^1^, ^3^, ^4^, ^5^ ^]^ Cancer‐associated fibroblasts (CAFs) are major constituents of the PDAC TME and can be categorized into functionally distinct cell populations, including myofibroblastic CAFs (myCAFs), inflammatory CAFs (iCAFs), antigen‐presenting CAFs (apCAFs), etc.^[^ ^5^, ^7^, ^8^ ^]^ Different from classical thought of CAFs as “immune neutral” cells, iCAFs are currently recognized as an important class of immune regulatory cells.^[^ ^7^, ^9^ ^]^ iCAFs, a critical subset of CAFs in PDAC, were identified by *Öhlund et al*. in 2017.^[^ ^7^, ^10^, ^11^ ^]^ In contrast to myCAFs, iCAFs are characterized by low α‐SMA expression and high secretion of inflammatory cytokines such as IL‐6, CCL2, and CXCL12.^[^ ^7^, ^10^, ^11^ ^]^ This distinct secretory profile enables iCAFs to recruit and modulate diverse immune cell populations, fostering an immunosuppressive microenvironment that may contribute to the poor response of PDAC to immunotherapy.^[^ ^7^ ^]^

Emerging evidence suggests that CAF phenotypes are highly dynamic and capable of interconversion, depending on spatial and biochemical cues within the PDAC microenvironment.^[^ ^7^, ^11^, ^12^, ^13^, ^14^, ^15^, ^16^, ^17^ ^]^ Various factors, including IL‐1,^[^ ^11^ ^]^ IL‐17A,^[^ ^18^ ^]^ FGF19,^[^ ^17^ ^]^ and circCUL2,^[^ ^13^ ^]^ have been identified to induce the inflammatory CAF phenotype and contribute to a poor prognosis in PDAC. Conversely, inhibition of the Hedgehog pathway has been shown to decrease myCAF population while concurrently augmenting iCAF population, resulting in a heightened immunosuppressive microenvironment.^[^ ^12^ ^]^ These findings highlight the potential of targeting iCAF induction and transformation as a strategy to enhance immunotherapy efficacy in PDAC. Recent studies have demonstrated that combining MEK and STAT3 inhibitors with anti‐PD‐1 therapy can attenuate the iCAF population and reprogram the immune microenvironment of PDAC, leading to improved antitumor responses and survival outcomes in both preclinical models and patients.^[^ ^16^ ^]^ However, many currently identified iCAF inducers act on immune pathways throughout the body, limiting their specificity and potentially causing systemic immune dysregulation. There is a need to identify more targeted approaches that specifically modulate iCAFs within the pancreatic TME.

In this study, we analyzed single‐cell RNA sequencing (scRNA‐seq) data from 59 PDAC patients to identify potential iCAF inducers secreted by cancer cells. Through a series of in vitro and in vivo experiments, we validated pancreatic polypeptide (PPY) as a potent inducer of the iCAF phenotype. PPY, a secretory protein predominantly expressed in the pancreas, was found to significantly promote an immunosuppressive microenvironment in PDAC mice models and patient samples. Importantly, targeting PPY enhanced the efficacy of anti‐PD‐1 immunotherapy in KPC (*Kras* ^LSL‐G12D/+^; *Trp53* ^LSL‐R172H/+^; *Pdx1*‐Cre) mice, as evidenced by reduced tumor volume on ^18^F‐FDG PET‐CT imaging and prolonged overall survival. Our findings provide insight into a novel mechanism of iCAF induction in PDAC and provide a more specific target for combination regimens of PDAC immunotherapy through blocking key cell communications in TME.

## 2 Results

### 2.1 The scRNA‐seq Analysis Reveals Molecules Associated with iCAF Induction

**Figure 1:**
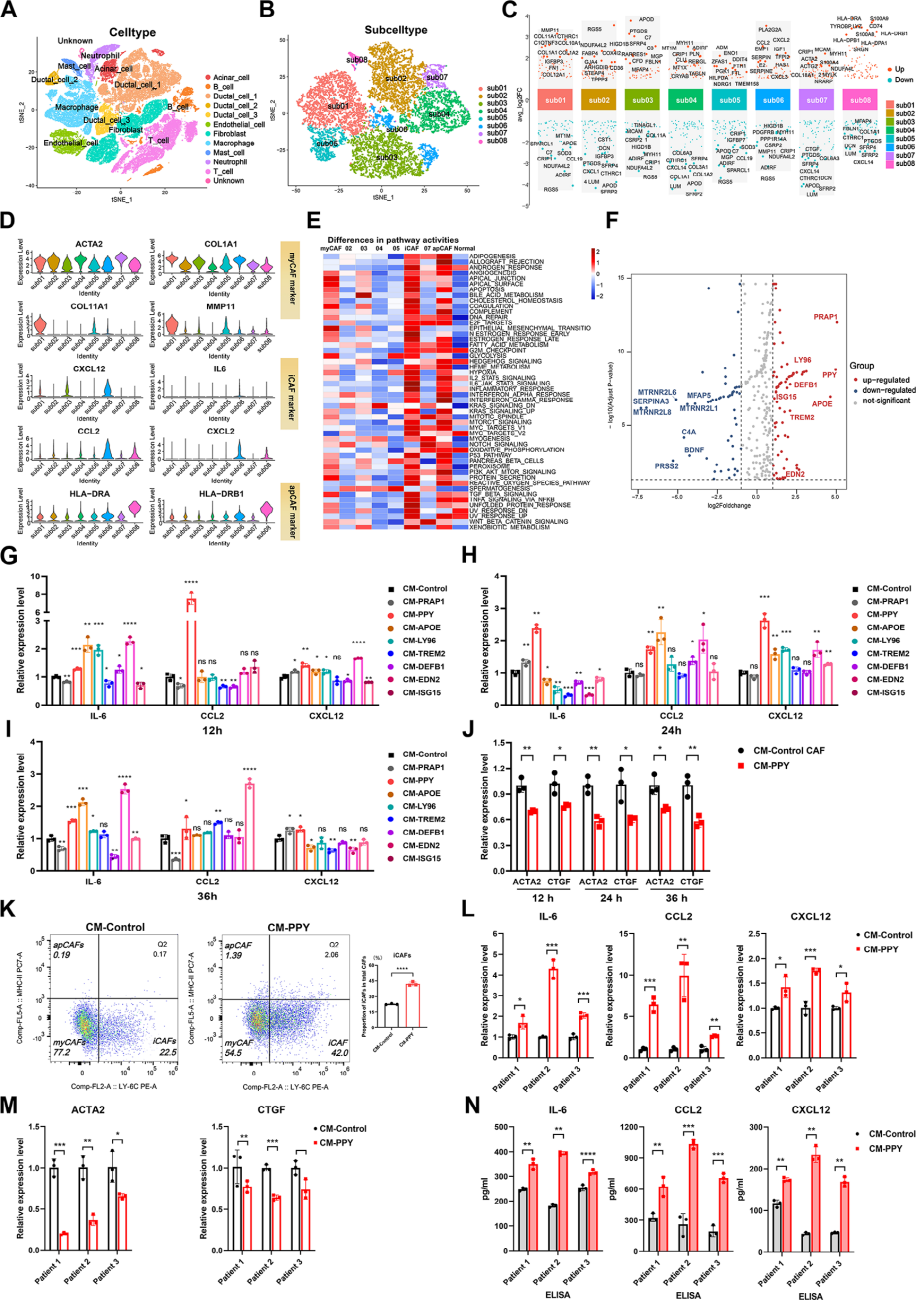
Screening and initial validation of cancer cell‐secreted proteins capable of significantly inducing iCAF phenotype. A) The t‐distributed stochastic neighbor embedding (t‐SNE) plot of the 92,222 cells in the single‐cell sequencing profile revealed distinct cell types observed in PDAC. B) The t‐SNE plot exhibited diverse subtypes of fibroblasts observed in PDAC. C) The top 10 up‐regulated and down‐regulated expressed marker genes of each CAF subgroup. D) The expression levels of myCAF markers (ACTA2, COL1A1, COL11A1, MMP11), iCAF markers (CXCL12, IL‐6, CCL2, CXCL2), and apCAF markers (HLA‐DRA, HLA‐DRB1) in different fibroblast subsets. E) Pathway activities scored by GSVA between different fibroblast subsets. F) The volcano plot depicts the differential expression of genes encoding secreted proteins in cancer cells derived from patients with high versus low iCAF. G–I) qRT‐PCR analysis was conducted to assess alterations in the expression levels of iCAF markers (IL‐6, CXCL12, and CCL2) in CAFs isolated from KPC mice following treatment with conditioned medium (CM) containing potential candidates for 12 (G), 24 (H), and 36 h (I). J) qRT‐PCR analysis of changes in the expression levels of myCAF markers (ACTA2 and CTGF) in CAFs isolated from KPC mice after treating them with CM containing PPY for 12, 24, and 36 h. K) Flow cytometry analysis of iCAF (Ly6C+MHC‐II‐), myCAF (Ly6C+MHC‐II‐), and apCAF (Ly6C+MHC‐II‐) populations after treating CAFs with CM containing PPY for 24 h. L,M) The changes in expression of iCAF markers (IL‐6, CXCL12, and CCL2) (L) and myCAF markers (ACTA2 and CTGF) (M) in CAFs isolated from three patients with PDAC were quantified by qRT‐PCR; following a 24‐h treatment with CM containing PPY. N) After treating CAFs derived from three PDAC patients with CM containing PPY for 24 h, ELISA was performed to assess the secretion of IL‐6, CCL2, and CXCL12. Each experiment was performed three times independently, and Student's *t*‐test was used to analyze the data. The results are presented as mean ± SD; *, *p* < 0.05; **, *p* < 0.01; ***, *p* < 0.001; ***, *p* < 0.001; ns, not statistically significant.

The immunosuppressive effects of inflammatory cancer‐associated fibroblasts (iCAFs) have been well‐documented across multiple human malignancies, including pancreatic cancer,^[^ ^11^, ^16^ ^]^ colon cancer,^[^ ^17^, ^19^, ^20^ ^]^ and esophageal cancer,^[^ ^21^, ^22^ ^]^ with clear correlations to patient survival and treatment resistance.^[^ ^11^, ^16^, ^19^, ^20^, ^21^, ^22^ ^]^ In this study, the results of survival analysis using data from The Cancer Genome Atlas (TCGA) further demonstrated that PDAC patients with high iCAF signature had shorter survival times, consistent with findings reported in the literature (Figure S1A, Supporting Information). To better understand iCAF regulation in PDAC and identify potential therapeutic targets, we conducted a comprehensive analysis of single‐cell RNA sequencing (scRNA‐seq) data from 59 PDAC patients, sourced from multiple databases (GEO: GSE111672, GSE154778, GSE155698, GSE154405; GSA: CRA001160). After quality filtering, gene expression normalization, and batch effect correction, we performed Principal Component Analysis (PCA) on genes variably expressed across 92,222 cells. Subsequently, we classified and annotated cell populations based on their marker gene expression (**Figure** **1**A; Figure S1B,C, Supporting Information). In addition to ductal cells, we identified fibroblasts, immune cells, endothelial cells, acinar cells, and epithelial cells (Figure 1A; Figure S1B,C, Supporting Information). The fibroblasts derived from PDAC samples were further classified into 8 distinct subclusters, and the heatmap depicting their expression profiles revealed each subcluster exhibiting a distinguished transcriptional profile and stromal component repertoire (Figure 1B,C; Figure S1D,E, Supporting Information). Three common CAF clusters of PDAC were identified: myCAFs (sub01; COL1A2, ACTA2, MMP11), iCAFs (sub06; IL‐6, CCL2, CXCL12), and apCAFs ((sub08; HLA‐DRA, HLA‐DRB) (Figure 1D). In addition, the scRNA‐seq data of normal pancreas acquired from the GEO database (accession numbers: GSE165399 and GSE81547) and the ArrayExpress database (accession numbers: E‐MTAB‐5061) was processed using similar processing steps, and normal fibroblast population was then isolated (Figure S1F,G, Supporting Information).

To characterize the functional properties of these CAF subsets compared to the normal fibroblast population, Gene set variation analysis (GSVA) was employed. This analysis revealed enhanced activation of multiple immune‐related signaling pathways (such as IL‐6‐JAK‐STAT3 signaling and the inflammatory response) in the iCAF population compared to other CAF subsets and normal pancreatic fibroblasts, while myCAF population exhibited significant activation in apical junction and epithelial‐mesenchymal transition (Figure 1E). Additionally, CellChat analysis demonstrated significant signaling interactions between iCAFs and various immune cell types (Figure S1H, Supporting Information), supporting their role in shaping the immunosuppressive microenvironment of PDAC. Further analysis of intercellular communications revealed strong bidirectional signaling between the Ductal_cell_1 group and iCAFs, emphasizing the importance of cancer cell‐iCAF interactions in the tumor microenvironment (Figure S1H,I, Supporting Information). To further elucidate these interactions, we visualized the signaling pathways through which cancer cells communicate with iCAFs to identify potential specific molecular targets (Figure S1J, Supporting Information). However, the outcomes did not fully meet our expectations. Molecular pairs such as SPP1‐ITGA and PRSS3‐F2R, which have been frequently predicted and reported in the single‐cell sequencing literature on macrophages,^[^ ^23^, ^24^, ^25^ ^]^ were also predicted and ranked at the top of the list (Figure S1J, Supporting Information). We also identified adhesion‐related molecules like LAMA3 and LAMB3,^[^ ^26^, ^27^ ^]^ which diverged from our primary research objectives (Figure S1J, Supporting Information). More importantly, the results from the CellChat analysis were constrained by the database's reliance on previously reported molecular pairs, potentially overlooking highly expressed but unreported pairs.

To identify principal iCAF inducers in the PDAC microenvironment, we further analyzed the single‐cell sequencing data from PDAC patients. Initially, patients were stratified into two groups based on the abundance and proportion of iCAFs: those in the top quartile were categorized into the high iCAF abundance group, while the remainder were assigned to the low iCAF abundance group (Figure S2A, Supporting Information). After filtering out normal cells through copy number variation (CNV) analysis, we conducted differential expression analysis of cancer cells derived from high‐iCAFs and low‐iCAFs groups (Figure S2B,C, Supporting Information). Considering that secreted protein‐receptor interaction is a predominant mode of intercellular communication and the accessibility of targeted therapy, we then utilized the HPA database to further identify genes responsible for encoding secreted proteins among these differentially expressed genes (Figure 1F). We identified eight secretory proteins that exhibited significantly elevated expression levels in cancer cells from the high‐iCAFs group, which were also associated with poor PDAC prognosis (Figure 1F; Figure S2D–K, Supporting Information). Among them, although the survival analysis of TREM2 yielded a p‐value greater than 0.05, a clear trend was observed (Figure S2H, Supporting Information). Given the critical role of TREM2 in chronic inflammation and cancer immunosuppression,^[^ ^28^, ^29^ ^]^ it was included for further analysis.

To identify the most potent molecules that induce iCAF transformation, plasmids highly expressing each of these 8 molecules were constructed and separately transfected into HEK 293T cells to obtain conditioned media (CM) containing a substantial amount of the candidate protein (Figure S3A, Supporting Information). Human CAFs and murine CAFs were respectively isolated from pancreatic cancer tissues from PDAC patients and KPC (*Kras* ^LSL‐G12D/+^; *Trp53* ^LSL‐R172H/+^; *Pdx1*‐Cre) mice, and the purity of these CAFs was assessed based on their morphology and immunohistochemical staining for FAP (labeled in red) and α‐SMA (labeled in green) prior to subsequent experiments (Figure S3B,C, Supporting Information). To mitigate the impact of individual patient variability on our findings, we initially employed these CM to treat CAFs from KPC mice (transgenic mouse model) for varying durations (12, 24, and 36 h) (Figure 1G–I). Among the candidates, pancreatic polypeptide (PPY) emerged as the most potent iCAF inducer. The qRT‐PCR analysis revealed an up‐regulation in the expression of iCAF markers (IL‐6, CCL2, and CXCL12) after treating CAFs with CM‐PPY for 12 h, with CCL2 exhibiting the most significant increase (Figure 1G). Moreover, the expression of IL‐6, CCL2, and CXCL12 was significantly enhanced after 24 h of CM‐PPY treatment, which persisted until 36 h post‐treatment (Figure 1H,I, Supporting Information). Meanwhile, we also investigated the expression of myCAF markers (ACTA2 and CTGF) that were reported in other studies,^[^ ^11^, ^30^ ^]^ and there was a significant decrease in their expression upon treatment with CM‐PPY (Figure 1J). Flow cytometry analysis confirmed a substantial increase in the iCAF proportion and a decrease in the myCAF proportion following CM‐PPY treatment (Figure 1K). To validate these findings in human cells, we treated CAFs isolated from three PDAC patients with CM of human PPY for 24 h. Consistent with the findings in KPC CAFs, PPY significantly induced the iCAF phenotype while suppressing myCAF characteristics in human CAFs (Figure 1L–N).

These findings suggest that PPY may play a pivotal role in inducing iCAFs in PDAC, which contributes to the development of an immunosuppressive microenvironment. Therefore, it is imperative to establish a comprehensive research framework for investigating this molecule.

### 2.2 PPY Induces iCAFs and Correlates with Poor Prognosis in Pancreatic Cancer

Pancreatic polypeptide (PPY), a member of the neuropeptide Y (NPY) family, is known to inhibit gastric and intestinal function as well as insulin action.^[^ ^31^ ^]^ Growing evidence has highlighted the association between aberrant gastrointestinal hormone secretion and PDAC development and progression.^[^ ^32^, ^33^, ^34^ ^]^ Recently, elevated PPY levels have been linked to increased Ki67 staining in pancreatic cancer tissue, suggesting a role in PDAC progression.^[^ ^35^ ^]^ However, the specific role of PPY in shaping the tumor immune microenvironment and its effects on CAFs remained unexplored.

**Figure 2:**
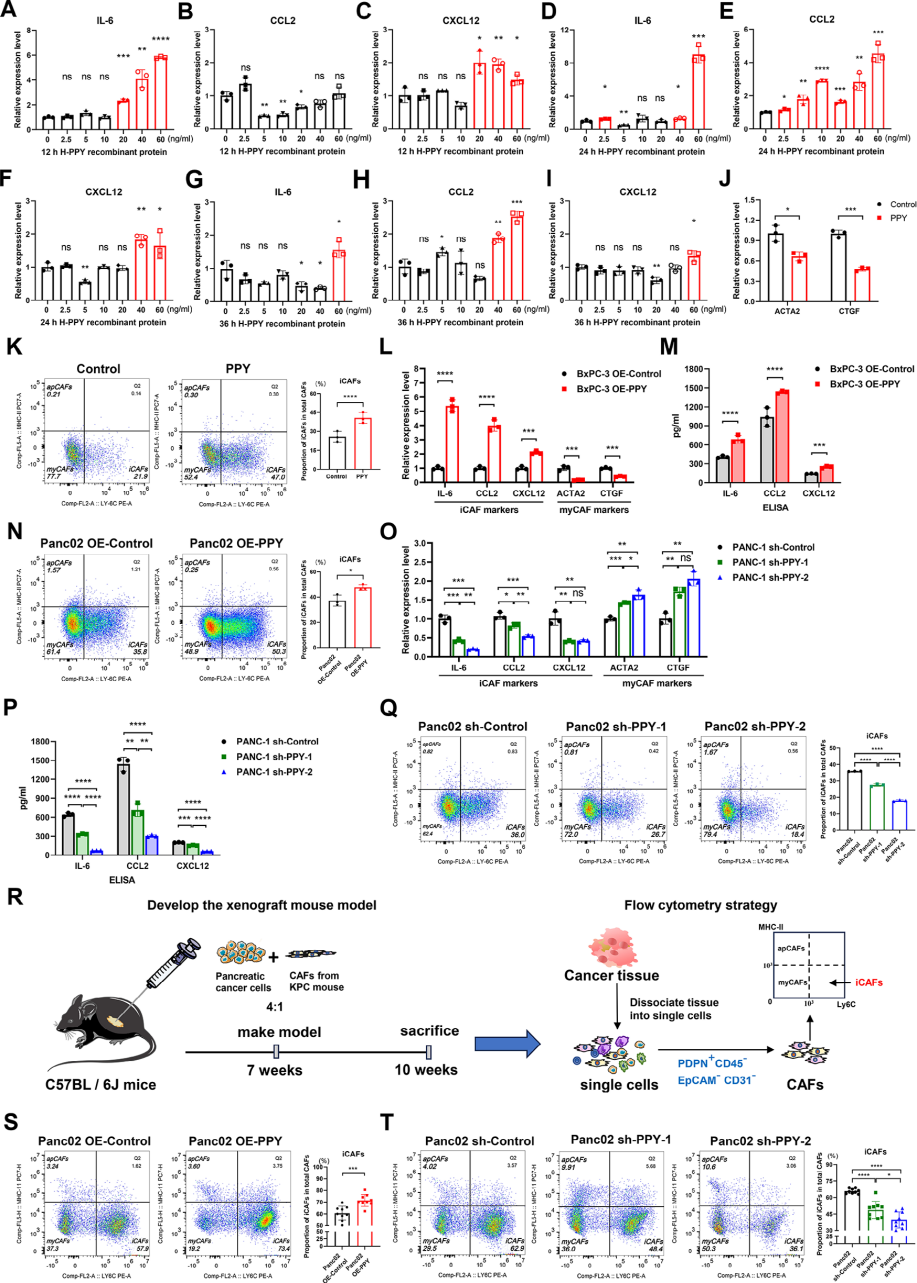
PPY significantly induces the iCAF phenotype in PDAC CAFs both in vitro and in vivo. A–C) After treating CAFs derived from human PDAC tissues for 12 h, qRT‐PCR analysis was performed to assess their alterations in the expression of iCAF markers (CXCL12 (A), IL‐6 (B), CXCL12 (C)). D–F) qRT‐PCR analysis of iCAF markers (CXCL12 (D), IL‐6 (E), CXCL12 (F)) after treating the human CAFs for 24 h. G–I) qRT‐PCR analysis of iCAF markers (CXCL12 (G), IL‐6 (H), CXCL12 (I)) after treating the human CAFs for 36 h. J) qRT‐PCR analysis of the expression levels of myCAF markers (ACTA2 and CTGF) after treating the human CAFs with PPY proteins (40ng/ml) for 24 h. K) Flow cytometry analysis was performed to evaluate the populations of iCAFs (Ly6C+MHC‐II‐), myCAFs (Ly6C+MHC‐II‐), and apCAFs (Ly6C+MHC‐II‐), after treating CAFs derived from cancer tissues of KPC mice with PPY recombinant proteins. L,M) After co‐culturing the human CAFs together with BxPC‐3 cells overexpressing PPY, the expression levels of iCAF markers (IL‐6, CCL2, and CXCL12) and myCAF markers (ACTA2 and CTGF) were quantified using qRT‐PCR (L), and the secretion levels of IL‐6, CCL2, and CXCL12 were measured using ELISA (M). N) The CAFs derived from cancer tissues of KPC mice were cocultured with Panc02 overexpressed PPY, and flow cytometry was applied to analyze iCAF, myCAF, and apCAF populations. O,P) After co‐culturing the human CAFs with PANC‐1 cells that had down‐regulated PPY expression, the expression levels of iCAF markers (IL‐6, CCL2, and CXCL12) and myCAF markers (ACTA2 and CTGF) were analyzed by qRT‐PCR (L), and secretion levels of IL‐6, CCL2, and CXCL12 were assessed by ELISA (M). Q) The murine CAFs were cocultured with Panc02 that had down‐regulated PPY expression, and flow cytometry was applied to analyze iCAF, myCAF, and apCAF populations. R) Schematic diagram of co‐injection of mouse cancer cells and CAFs (4:1) derived from KPC mice to construct the orthotopic allograft tumor model in C57BL/6J mice (n = 8), and the tumor tissues were isolated and analyzed by flow cytometry. S,T) The tumor tissues of PPY upregulated and downregulated groups and their respective control groups were dissociated into single cells, and flow cytometry was utilized to analyze the iCAF, myCAF, and apCAF populations in the tumor tissue. Student's *t*‐test was used to analyze the data, and the results are presented as mean ± SD; *, *p* < 0.05; **, *p* < 0.01; ***, *p* < 0.001; ***, *p* < 0.001; ns, not statistically significant. OE, overexpression.

To further validate the ability of PPY to induce iCAFs, we treated CAFs derived from PDAC patients and KPC mice with human and mouse PPY recombinant proteins, respectively, at various time points and concentrations (**Figure** **2**A–K; Figure S3D–J, Supporting Information). After treating human CAFs with human PPY protein at concentrations of 20 ng mL^−1^ or higher concentrations for 12 h, there was a significant up‐regulation in the expression of IL‐6 and CXCL12 (Figure 2A–C). Following 24 h of the treatment, human CAFs treated with various concentrations of PPY showed a significant increase in CCL2 expression, while human CAFs treated with 40 ng mL^−1^ or higher concentrations of human PPY continued to exhibit strong promotion of IL‐6 and CXCL12 expression (Figure 2D–F). Furthermore, even after 36 h of treatment, CAFs treated with 60 ng mL^−1^ human PPY protein demonstrated sustained effectiveness in promoting the expression of iCAF markers (IL‐6, CCL2, CXCL12) (Figure 2G–I). Similarly, murine PPY treatment (10 ng mL^−1^) for 12 and 24 h resulted in a significant upregulation of iCAF markers in murine CAFs (Figure S3D–I, Supporting Information). The expression levels of myCAF markers (ACTA2 and CTGF) and the proportion of myCAFs were found to decrease, while the proportion of iCAFs increased, as demonstrated by qRT‐PCR and flow cytometry analyses (Figure 2J,K; Figure S3J, Supporting Information). The induction effect of PPY exhibited a slow but persistent characteristic and was highly dependent on the concentration of PPY. Higher concentrations of PPY were required to effectively stimulate the upregulation of iCAF markers over time, potentially due to the consumption of PPY protein. This observation also suggests that the induction process of iCAFs by PPY is reversible, providing an opportunity to prevent the formation of iCAFs through targeted intervention. In addition, we also examined the iCAF promotion effect of IL‐1, a well‐known iCAF stimulant, with the concentrations that were reported in other studies for different durations.^[^ ^11^ ^]^ The qRT‐PCR analysis showed that IL‐1 significantly increased the expression of IL‐6 and CCL2, but made little difference in the expression of CXCL12 expression, a key mediator of the immunosuppressive microenvironment of PDAC (Figure S3K–P, Supporting Information). These findings were consistent with previous reports.^[^ ^36^ ^]^ The promotion effect of IL‐1 on the expression of IL‐6 and CCL2 reached the peak rapidly (4 h) and subsequently declined, but the effects in human CAFs recovered at 24 h post‐treatment. (Figure S3K–P, Supporting Information). Given that previous studies have reported that IL‐1 treatment strongly induces IL‐1 expression in iCAFs,^[^ ^11^, ^13^ ^]^ the observed reduction in this promotion is unlikely attributable to IL‐1 consumption. Instead, it may more plausibly be explained by the establishment of a negative feedback loop.

**Figure 3:**
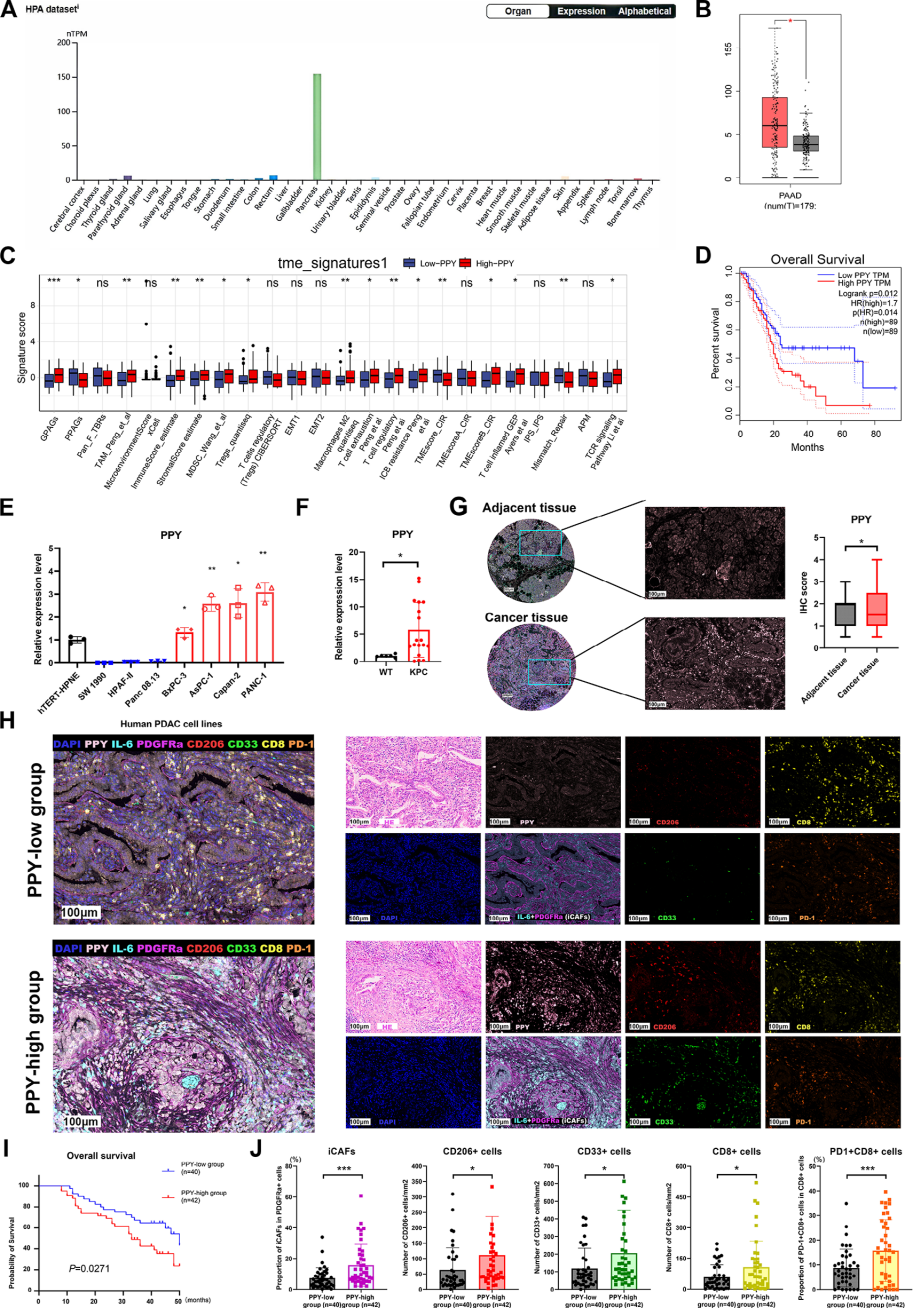
The expression characteristics of PPY in PDAC and its correlation with the immune landscape and clinical prognosis of PDAC. A) The HPA database showed the expression of PPY in various organs. B) the different expression levels of PPY in cancer tissues (red) and non‐tumor tissues (black) were analyzed by the GEPIA database. C) The correlation between PPY expression and TME signature was analyzed using IOBR tool. D) The correlation between PPY expression and the overall survival (OS) was analyzed by the GEPIA database. E) qRT‐PCR analysis of PPY expression in human PDAC cell lines. F) qRT‐PCR analysis of PPY expression in wild type (WT) C57BL/6J mice and KPC mice. G) mIHC was applied to analyze the PPY expression in the paired tumor tissue and adjacent tissue of PDAC (n = 82). The statistical data is presented as mean ± SD and analyzed using the paired t‐test. *, *p* < 0.05. H) The multiplexed immunohistochemistry (mIHC) technique was employed to examine the correlation between PPY expression and iCAF density, as well as immune cell infiltration in PDAC tissues from PPY‐low and PPY‐high groups in a PDAC tissue microarray. The staining scheme was as follows: PPY (pink), iCAFs (IL‐6, cyan; PDGFRa, magenta), CD206^+^cells (red), CD33^+^cells (green), CD8^+^T cells (CD8, yellow), PD‐1^+^cells (orange). All scale bars, 100 µm. I) Kaplan‐Meier survival plot depicts overall survival of patients in PPY‐low group and PPY‐high group. J) Summary and statistical analysis of the density of iCAFs and infiltration of CD206+cells, CD33+cells, CD8^+^T cells, and PD‐1^+^CD8^+^T cells in cancer tissues between the PPY‐low group (n = 40) and the PPY‐high group (n = 42). Statistics are shown in mean ± SD and accessed by the unpaired *t*‐test. *, *p* < 0.05; ***, *p* < 0.001. mIHC, multiplexed immunohistochemistry.

According to the qRT‐PCR analysis of PPY expression in pancreatic cancer cell lines, PANC‐1 cells exhibiting high levels of PPY, BxPC‐3 cells with low levels of PPY, and murine pancreatic cancer cell line Panc02 were selected for further investigation (**Figure** **3**E). And lentivirus was employed to modulate their expression of PPY, respectively. Upregulating PPY expression in cancer cells was found to increase the expression of iCAF markers in human and murine CAFs and the proportion of iCAFs, accompanied by decreased expression of myCAF markers and myCAF proportion (Figure 2L–N; Figure S4C,D, Supporting Information). Conversely, downregulating PPY expression in cancer cells resulted in a decrease in the proportion of iCAFs and their expression of iCAF markers, accompanied by an increased proportion of myCAFs and expression of myCAF markers (Figure 2O–Q; Figure S4G,H, Supporting Information). Additionally, blocking PPY with an antibody in the co‐culture system also impaired cancer cells' ability to induce iCAFs (Figure S4I–M, Supporting Information). To validate these findings in vivo, we established an orthotopic allograft mouse model of PDAC in C57BL/6 mice using cancer cells with regulated PPY expression and CAFs derived from KPC mice. At the experimental endpoint, tumor tissues were collected and dissociated for flow cytometry analysis. A significant increase in the proportion of iCAFs among total CAFs was found in the PPY‐overexpressing group, while this proportion decreased in the PPY knockdown group (Figure 2R–T).

Given the significant role of PPY in iCAF induction, we further investigated its potential as a therapeutic target in PDAC. Analysis of the Human Protein Atlas (HPA) dataset revealed predominant PPY expression in pancreatic tissue (Figure 3A), suggesting potential therapeutic specificity with minimal off‐target effects. Both HPA and Gene Expression Profiling Interactive Analysis (GEPIA) databases demonstrated significant upregulation of PPY expression in PDAC tissues compared to normal pancreatic tissues (Figure 3B; Figure S5A, Supporting Information). Single‐cell sequencing analysis revealed distinct PPY expression patterns across cell populations. In pancreatic cancer tissues, (Figure S5B,C, Supporting Information). In PDAC tissues, PPY expression was highest in Acinar_cell and Ductal_cell_1 groups (Figure S5B, Supporting Information). In normal tissue, PPY expression was minimal in ductal cells but detected in α_cell_2 and β_cell_2 populations (Figure S5C, Supporting Information). However, PPY was predominantly expressed in pancreatic PP cells. To further investigate this, we evaluated the expression levels of specific molecular markers for the α‐cell (GCG), β‐cell (INS), and δ‐cell (SST) populations. The results demonstrated that the expression levels of marker genes in this specific cell population were significantly higher compared to other cell populations. (Figure S5D–F, Supporting Information), indicating the accuracy of the annotation information. The apparent discrepancy in endocrine cell expression patterns may reflect similarities among endocrine cells and technical limitations in resolving rare PP cell populations. Notably, PDAC ductal cells showed markedly elevated PPY expression compared to normal ductal cells (Figure S5G, Supporting Information). Pseudotime trajectory analysis of ductal cells of PDAC utilizing Monocle2 revealed nine distinct states, with PPY expression progressively increasing during cancer progression (Figure S5H–M, Supporting Information). GEPIA database analysis further demonstrated that elevated PPY expression correlates with reduced overall survival in PDAC patients (Figure 3D). The qRT‐PCR experiments confirmed high expression levels of PPY across multiple human pancreatic cell lines and significantly higher expression in pancreatic tissues from KPC mice compared to wild‐type mice (Figure 3E,F).

To comprehensively characterize PPY expression and the immune landscape within the PDAC microenvironment, we conducted comprehensive bioinformatic and histological analyses. IOBR analysis revealed significant positive correlations between PPY expression and multiple immunosuppressive cell populations, including tumor‐associated macrophages (TAMs), myeloid‐derived suppressor cells (MDSCs), M2 macrophages, exhausted T cells, and regulatory T cells (Tregs) (Figure 3C). Furthermore, PPY expression exhibited a positive correlation with various iCAF subtypes, including detox_iCAFs, IL_iCAFs, and IFNγ_iCAFs (Figure S6A, Supporting Information). Furthermore, we performed multiplexed immunohistochemistry (mIHC) on a tissue microarray comprising 82 pairs of PDAC cancer tissues and adjacent normal tissues. The results showed significantly higher expression levels of PPY in cancer tissues compared to adjacent normal tissues, which were negatively correlated with patient survival prognosis (Figure 3G,I). In tissues exhibiting high PPY expression levels, there was an increased proportion of iCAFs and elevated infiltrations of immunosuppressive cells, including myeloid cells (CD33^+^ cells) and CD206^+^ cells (a marker commonly used to label M2 macrophages) (Figure 3H,J). In addition, CD8^+^ T cells in these tissues exhibited lower infiltration rates but increased PD‐1 expression, indicative of an exhausted phenotype (Figure 3H,J). Patients were stratified into two groups according to the median iCAF proportion, with those in the high‐iCAF group exhibiting significantly shorter overall survival (OS) times (Figure S6G, Supporting Information). Single‐cell sequencing data were also utilized to investigate the relationship between PPY expression and immune landscape, the results were consistent with the above findings (Figure S6C–F, Supporting Information).

Collectively, these results suggest that PPY induces the formation of iCAFs both in vivo and in vitro, and the expression of PPY is closely associated with the establishment of an immunosuppressive microenvironment and poor prognosis in PDAC.

### 2.3 PPY‐Induced iCAFs Reshape PDAC Immune Microenvironment and Promote Cancer Progression

**Figure 4:**
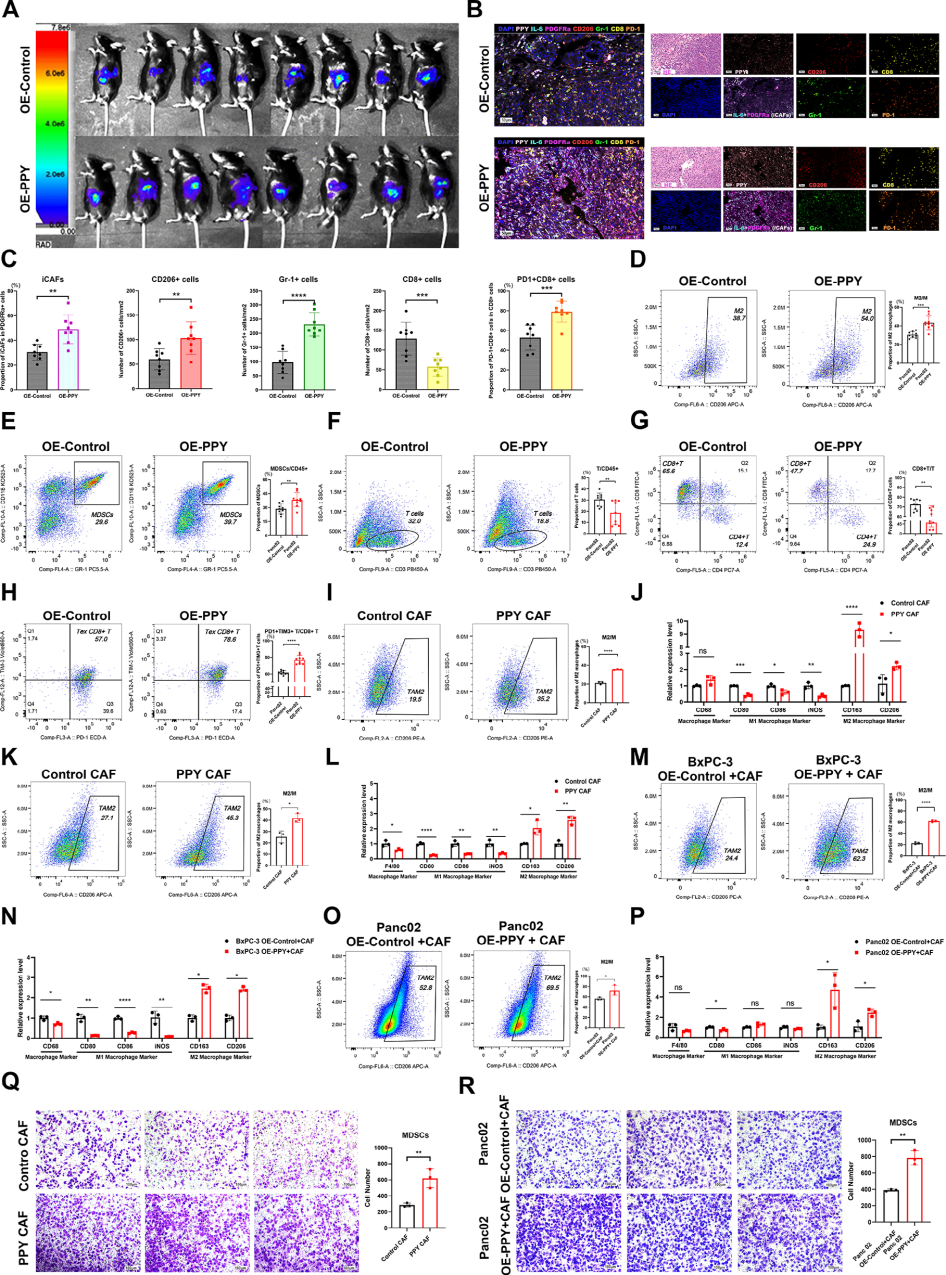
PPY‐induced iCAFs cultivate an immunosuppressive microenvironment of PDAC and promote cancer progression. A) After upregulating PPY expression in mouse cancer cells using recombinant lentivirus, an orthotopic allograft tumor model was established by conjecting these cancer cells and KPC mouse‐derived CAFs with a 4:1 ratio (n = 8). 20 days later, the vivo imaging system (IVIS) was applied to assess the tumor growth. B) mIHC staining of PPY (pink), iCAFs (IL‐6, cyan; PDGFRa, magenta), M2 macrophages (CD206, red), MDSCs (Gr‐1, green), CD8^+^T (CD8, yellow), PD‐1^+^ cells(orange) in TME of model mice. Scale bars, 50 µm. C) Summary and statistical analysis of PPY expression and the density of iCAFs and immune cells analyzed by mIHC in the TME of cancer tissues. D–H) Flow cytometry analysis of M2 macrophages (D), MDSCs (E), T cells (F), CD8^+^ T cells (G), exhausted CD8^+^T (H) in the TME of PPY overexposing group and respective control group. I–L) After co‐culturing human macrophages derived from THP‐1 cells (I and J) and mouse macrophages isolated from WT C57BL/6J mice (K and L) with CAFs treated with PPY recombinant proteins respectively, flow cytometry (I and K) and qRT‐PCR (J and L) were applied to analyze the M2 macrophage polarization in these macrophages. M–P) Flow cytometry (M and O) and qRT‐PCR (N and P) were applied to analyze the M2 macrophage polarization in human (M and N) and mouse macrophages (O and P), after co‐culturing them with CAFs that treated with the supernatants from human and pancreatic cancer cells with upregulated PPY expression, respectively. Q,R) Transwell migration assays showed the migration abilities in MDSCs stimulated by pancreatic cancer cells treated with PPY protein (Q) or pancreatic cancer cells over‐expressing PPY (R). Scale bars, 100 µm. The statistical data is presented as mean ± SD and analyzed using the unpaired *t*‐test. *, *p* < 0.05; **, *p* < 0.01; ***, *p* < 0.001; ***, *p* < 0.001; ns, not statistically significant. TME, tumor microenvironment. MDSCs, myeloid‐derived suppressor cells.

**Figure 5:**
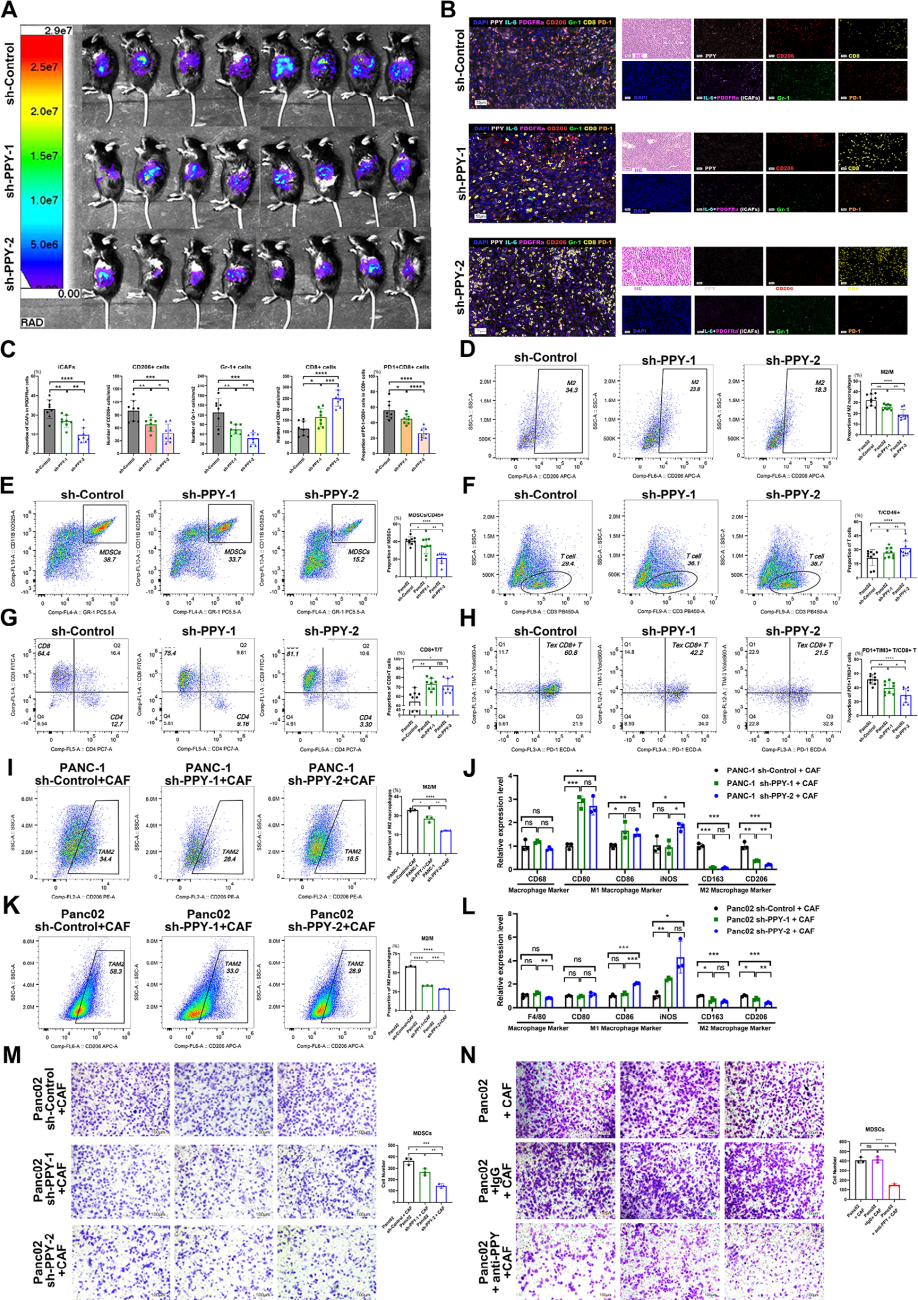
Down‐regulating PPY ameliorated the immunosuppression microenvironment of PDAC and decelerated cancer progression. A) Following down‐regulating PPY expression in mouse cancer cells using recombinant lentivirus, an orthotopic allograft tumor model was established by conjecting these cancer cells and KPC mouse‐derived CAFs with a 4:1 ratio (n = 8). 20 days later, a vivo imaging system (IVIS) was applied to monitor the tumor growth. B) mIHC staining of PPY (pink), iCAFs (IL‐6, cyan; PDGFRa, magenta), M2 macrophages (CD206, red), MDSCs (Gr‐1, green), CD8+T cells (CD8, yellow), PD‐1^+^cells (orange) in TME of model mice. Scale bars, 50 µm. C) Summary and statistical analysis of PPY expression and the density of iCAFs and immune cells analyzed by mIHC in the TME of cancer tissues. D–H) Flow cytometry analysis of M2 macrophages (D), MDSCs (E), T cells (F), CD8+ T cells (G), exhausted CD8+T (H) in the TME of the group of cancer cells with down‐regulated PPY expression and respective control group. I–L) The human macrophages derived from THP‐1 cells (I and J) and mouse macrophages isolated from WT C57BL / 6J mice (K and L) were respectively co‐cultured with CAFs, that were respectively treated with the supernatants from cancer cells with reduced PPY expression. Flow cytometry (I and K) and qRT‐PCR (J and L) were applied to analyze the M2 macrophage polarization in these macrophages. M) Transwell migration assays showed the migration abilities in MDSCs stimulated by pancreatic cancer cells with reduced PPY expression. N) Transwell migration assays showed the alterations in the migratory abilities of MDSCs when a PPY antibody was added to their co‐culture system with pancreatic cancers. Scale bars, 50 µm. Statistics are shown in mean ± SD accessed by the paired *t*‐test. *, *p* < 0.05; **, *p* < 0.01; ***, *p* < 0.001; ***, *p* < 0.001; ns, not statistically significant.

Building on our observations of PPY expression and immune landscape in the tissue microarray, we further investigated the effects of PPY‐induced iCAFs on the immune microenvironment of PDAC. Initially, we conducted a comprehensive analysis of immune cell infiltration in cancer tissues of the orthotopic allograft mouse model. The results of mIHC staining and flow cytometry revealed that the group with high PPY expression in cancer cells exhibited not only a significantly higher proportion of iCAFs but also increased presence of M2 macrophages and myeloid‐derived suppressor cells (MDSCs) within the TME compared to the control group (Figure 2S,; **Figure** **4**B–E). Additionally, there was a decreased infiltration of T cells, particularly CD8^+^ T cells, in the PPY‐overexpression group (Figure 4B,C; Figure 4F,G). Importantly, we noted enhanced infiltration of exhausted CD8^+^ T (Tex) cells in the PPY overexpression group (Figure 4H). Tex cells, characteristic of both cancers and chronic infections, exhibit elevated expression of inhibitory receptors (PD‐1, TIM‐3, LAG‐3, etc.), impaired effector functions, and limited proliferative potential.^[^ ^37^ ^]^ These cells exert only weak or temporary immune pressure on cancer cells, making their reversal a crucial target for immunotherapy strategies.^[^ ^37^, ^38^ ^]^ In contrast, down‐regulating PPY in cancer cells resulted in a decrease in the proportion of iCAFs within the TME, accompanied by reduced infiltration of M2 macrophages and MDSCs but increased levels of T cells (Figure 2T; **Figure** **5**B,F). Notably, the infiltration of CD8^+^ T cells was higher than that observed in the control group, with a concomitant decline in the proportion of Tex cells (Figure 5G,H). More importantly, when assessing tumor volume using the in vivo imaging system (IVIS), tumors in the PPY‐overexpression group exhibited larger sizes compared to those in the control group, while the PPY down‐regulated group showed smaller volumes compared to the control group (Figures 4A and 5A). These findings were consistent with gross images of extracted tumor tissues at the experimental endpoint (Figure S7A,B, Supporting Information).

To further elucidate the direct effects of PPY‐induced iCAFs on immune cells, we conducted in vitro co‐culture experiments. The primary bone marrow cells isolated from wild‐type C57BL/6J mice were treated with M‐CSF and GM‐CSF, respectively, to obtain murine macrophages and MDSCs. THP‐1 cells were stimulated with PMA to generate human macrophages. After sorting and purifying these cells using flow cytometry, they were utilized in subsequent experiments (Figure S7K–M, Supporting Information). We generated iCAFs by treating CAFs derived from PDAC patients and KPC mice with species‐specific PPY recombinant proteins, and these iCAFs were then co‐incubated with human and mouse macrophages, respectively. Flow cytometry and qR‐PCR analyses revealed that these PPY‐induced iCAFs promoted M2 polarization in both human and mouse macrophages (Figure 4I–L). Similar results were observed when iCAFs were generated using supernatants from cancer cells overexpressing PPY (Figure 4M–P). Conversely, CAFs treated with supernatants from cancer cells with reduced PPY expression or adding PPY antibody to the co‐culture system exhibited an attenuated ability to promote M2 polarization in these macrophages (Figure 5I–L; Figure S7I,J, Supporting Information). Furthermore, PPY‐induced iCAFs exhibited a stronger ability to promote MDSC migration compared to control CAFs, while the inhibition or blockade of PPY attenuated this ability (Figure 4Q,R; Figure 5M,N).

These results demonstrate that PPY‐induced iCAFs can cultivate an immunosuppressive environment in PDAC through polarizing macrophages toward the M2 phenotype and facilitating MDSC recruitment. These immunosuppressive cells may contribute to the decreased presence of CD8^+^T cells in the TME and their exhausted phenotype. Recent studies have shown that reactivation of Tex cells can be achieved by blocking PD‐1 or other inhibitory pathways, highlighting a potential avenue for anti‐tumor therapy.^[^ ^37^, ^38^ ^]^ Effective CD8^+^ T cell responses are critical for anti‐tumor immunity, which could explain the observed decrease in tumor volume upon PPY knockdown.

### 2.4 PPY Induces the iCAF Phenotype by Activating the Noncanonical NF‐κB Pathway Through EGFR

**Figure 6:**
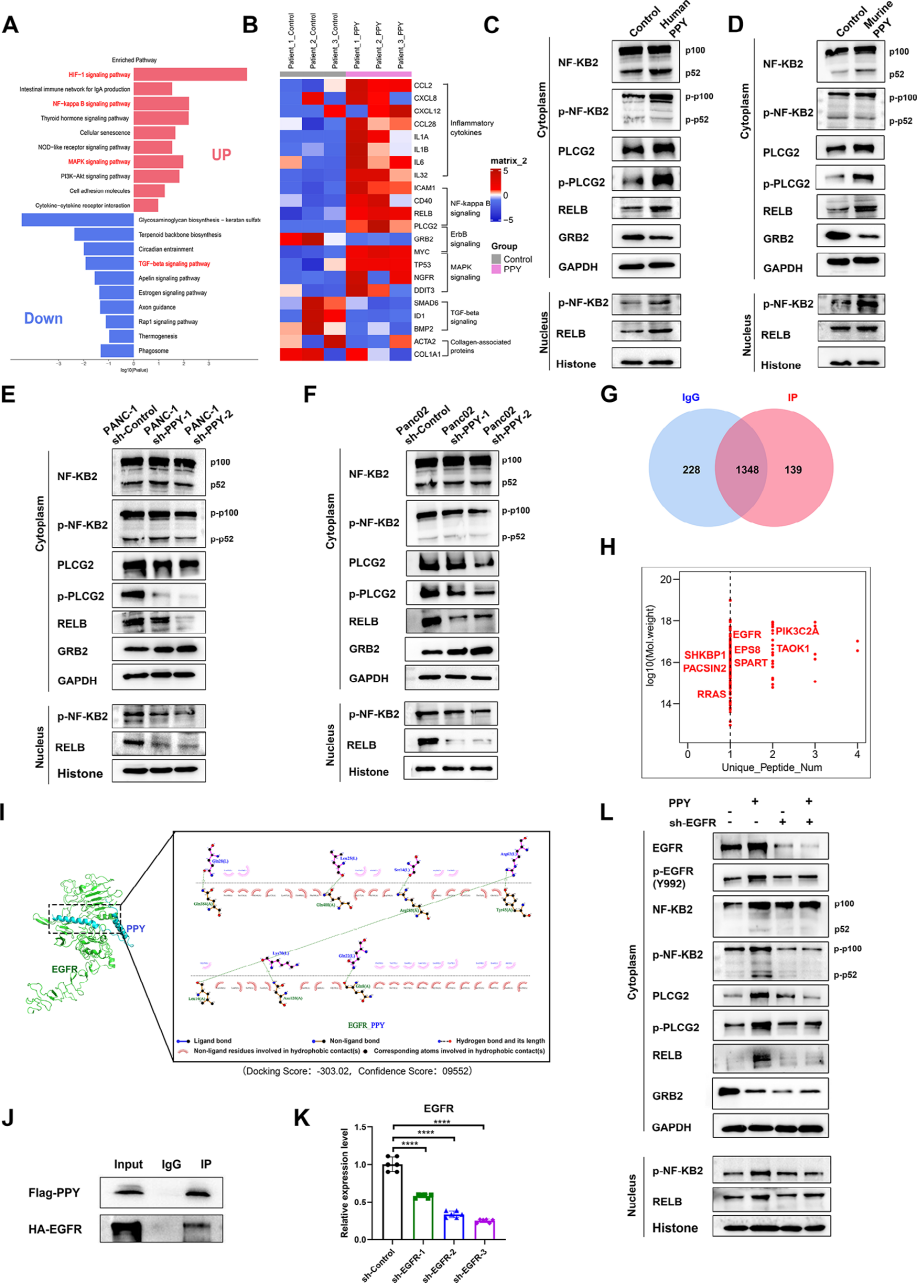
PPY induced iCAF phenotype by activating the NF‐κB2 pathway through EGFR. A) CAFs from 3 PDAC patients were treated with PPY recombinant proteins (40 ng/ml) for 24 h, and mRNA sequencing and KEGG pathway enrichment analysis were performed on these CAFs and responding control CAFs. The top ten up‐regulated and down‐regulated pathways were exhibited. B) The expression levels of molecules in the categories or pathways of our interest are displayed. C,D) Human CAFs (C) and KPC CAFs (D) were respectively treated with PPY recombinant proteins, and immunoblot analysis revealed their alterations in the expression of markers associated with the NF‐κB2 pathway and molecules involved in ERBB signaling. E,F) Human CAFs (E) and KPC CAFs (F) were respectively treated with the supernatants of cancer cells exhibiting down‐regulated PPY expression, and immunoblot analysis revealed their alterations in the expression of NF‐κB2 pathway markers. G) Venn diagram illustrating the different proteins detected by mass spectrometry in the IgG group and IP group. H) The unique proteins identified in the IP group were shown. I) Molecular docking results showed that PPY had a good binding interaction with the EGFR protein target conformation. J, Co‐immunoprecipitation assays showed the interaction between PPY and EGFR. K) The efficiency of EGFR knockdown in human CAFs was examined by qRT‐PCR. L) Immunoblot analysis revealed that downregulation of EGFR expression in human fibroblasts led to alterations in the expression of NF‐κB2 pathway markers upon PPY stimulation. The statistical data is presented as mean ± SD and analyzed using the paired *t*‐test. *, *P* < 0.05; **, *P* < 0.01; ***, *P* < 0.001.

To elucidate the molecular mechanism underlying PPY‐induced iCAF transformation, we conducted mRNA sequencing to estimate the expression changes in PPY‐conditioned CAFs derived from 3 patients with PDAC. KEGG pathway enrichment analysis of the altered genes revealed that the NF‐κB signaling pathway, a well‐established signature related to iCAF induction,^[^ ^7^, ^13^ ^]^ and the HIF‐1 signaling pathway, recently identified as an iCAF feature,^[^ ^39^ ^]^ were enriched in the top 10 upregulated pathways (**Figure** **6**A). In contrast, TGF‐β, a well‐known myCAF feature, was enriched in the top 10 down‐regulated pathways (Figure 6A). Further analysis of the molecules enriched in these pathways showed significant upregulation of inflammatory cytokines, including IL‐6, CCL2, and CXCL12, in PPY‐treated CAFs, while the expression of collagen‐associated proteins decreased (Figure 6B). Notably, RELB, a pivotal molecule in the non‐canonical NF‐κB pathway (also known as the NF‐κB2 signaling pathway), and PLCG2, a molecule that facilitates RELB activation, exhibited significant upregulation (Figure 6B). In addition, it is noteworthy that the NF‐KB2 pathway exerts a slower but more sustained response compared to the NF‐KB1 pathway,^[^ ^40^ ^]^ aligning with our observation that iCAF marker expression changes manifested ≈12 h after PPY treatment (Figure 2A–I). Subsequently, we evaluated the activation of NF‐κB2 signaling in human and mouse CAFs treated with PPY. The administration of PPY proteins facilitated RELB expression, phosphorylation of NF‐κB2, and their translocation into the nucleus (Figure 6C,D). In contrast, these phenomena were inhibited when PPY expression was downregulated (Figure 6E,F).

To further elucidate how PPY initiates the activation of the NF‐κB2 signaling pathway and subsequent iCAF transformation, immunoprecipitation combined with mass spectrometry (IP‐MS) was employed to identify the specific receptor targeted by PPY. We generated a PPY‐Flag plasmid over‐expressing the predominant PPY splicing variant and enriched the protein using an ultrafiltration tube after transfecting the plasmid into HEK 293T cells. IP‐MS analysis was then performed on CAFs treated with the enriched PPY‐Flag supernatant. After excluding proteins pulled down in the IgG control group, a total of 138 unique proteins were identified in the IP group (Figure 6G,H). Notably, the epidermal growth factor receptor (EGFR) (also called ERBB1) and molecules involved in the EGFR signaling cascade, including RRAS, PIK3C2A, SPART, and PACSIN2, were detected (Figure 6H). Our mRNA‐seq data revealed upregulation of PLCG2 (encoding PLCγ) and downregulation of GRB2 within the ERBB signaling pathway (Figure 6B). EGFR, a receptor tyrosine kinase, can phosphorylate multiple downstream effectors,^[^ ^41^, ^42^ ^]^ and numerous studies have demonstrated its role in NF‐κB activation.^[^ ^42^, ^43^, ^44^, ^45^, ^46^ ^]^ Single‐cell RNA‐seq analysis revealed high EGFR expression in iCAFs (Figure S8A, Supporting Information). However, recent research has also implicated EGFR in myCAF function and pancreatic cancer metastasis.^[^ ^30^ ^]^ They found that EGFR/ERBB2 signaling was induced by TGF‐β in myCAFs through an autocrine process mediated by amphiregulin, and the phosphorylation of Y1068 was important during the process.^[^ ^30^ ^]^ While Y1068 phosphorylation primarily recruits GRB2 to activate MAPK signaling,^[^ ^42^ ^]^ our analyses showed that PPY treatment downregulated GRB2 expression while upregulating PLCG2 expression (Figure 6B–F). PLCγ, encoded by PLCG2, is closely associated with NF‐κB and other immune signaling pathways, and it is recruited and activated following phosphorylation at the Y992 site of EGFR.^[^ ^42^ ^]^ Based on these findings, we investigated the impact of PPY on EGFR Y992 phosphorylation. The results indicated that PPY facilitated EGFR Y992 phosphorylation, while the downregulation of PPY expression in cancer cells within co‐culture systems suppressed this phosphorylation (Figure S8H, Supporting Information–K). In addition, PPY exerted a minimal effect on EGFR expression, with only a slight increase observed in human CAFs treated with recombinant proteins (Figure 8D–G, Supporting Information). Bioinformatics analysis revealed a weak correlation between EGFR and PPY expression (Figure 8B,C, Supporting Information), indicating that PPY primarily influences EGFR activation rather than expression. These findings suggest that EGFR plays distinct roles in myCAF and iCAF signaling pathways depending on the activating stimulus. It may explain the observed downregulation of both myCAF and iCAF signatures in EGFR‐knockout pancreatic stellate cells co‐cultured with PDAC organoids.^[^ ^30^ ^]^ In addition, targeting EGFR has been reported to prolong the survival time in PDAC patients in a phase III clinical trial.^[^ ^47^ ^]^

**Figure 7:**
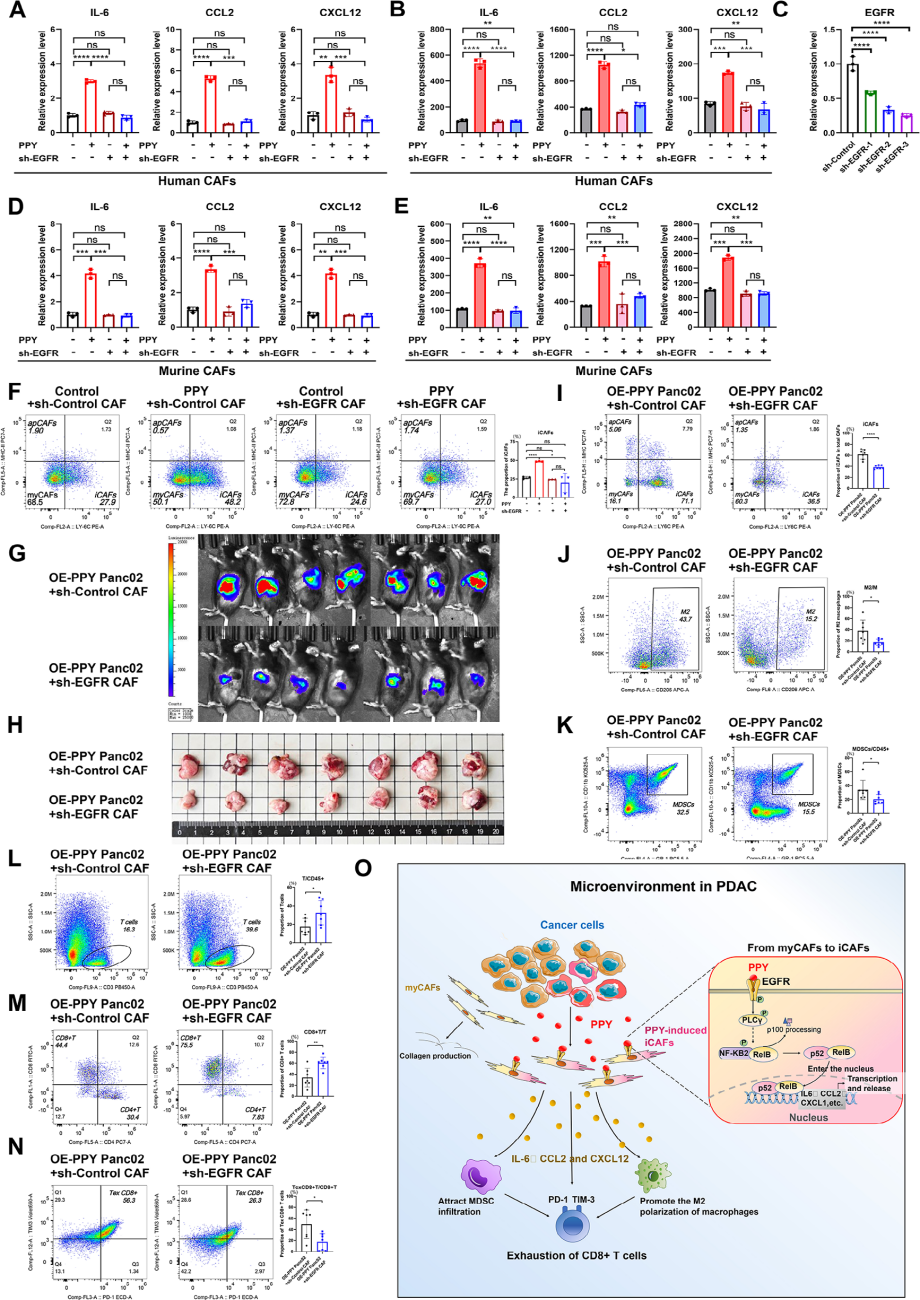
The inhibition of EGFR expression in CAFs impeded the induction of iCAFs by PPY. A) qRT‐PCR (A) and B) ELISA analyses of the expression levels of IL‐6, CCL2, and CXCL12 in human EGFR‐knockdown CAFs treated with PPY proteins. C) The efficiency of EGFR knockdown in KPC CAFs was examined by qRT‐PCR. D,E) qRT‐PCR (D) and ELISA (E) analyses of the expression levels of IL‐6, CCL2, and CXCL12 in murine EGFR knockdown CAFs treated with PPY proteins. F) Flow cytometry analysis was performed to evaluate the populations of iCAFs, myCAFs, and apCAFs in murine EGFR‐knockdown CAFs treated with PPY proteins. G,H) The IVIS image (G) and gross image (H) of tumors in model mice (n = 7), that was constructed by co‐injecting cancer cells with up‐regulated PPY expression and KPC CAFs with down‐regulated EGFR expression. I–N) Flow cytometric analysis was performed to evaluate the presence of iCAFs (I), M2 macrophages (J), MDSCs (K), T cells (L), CD8^+^T cells (M), and exhausted CD8^+^T cells (N) within the tumor microenvironment. O) Map of scientific hypotheses of this article. The statistical data is presented as mean ± SD and analyzed using the unpaired *t‐*test. *, *p* < 0.05; **, *p* < 0.01; ***, *p* < 0.001.

To further investigate the direct interactions between PPY and EGFR in human CAFs, we employed molecular docking to examine their conformational compatibility. The results showed a significant potential for PPY to directly bind with EGFR (Docking Score: −303.02; Confidence Score: 0.9552), as multiple amino acids within the extracellular domain of EGFR were found to possess the ability to form a binding pocket with PPY (Figure 6I). Furthermore, co‐immunoprecipitation (Co‐IP) experiments were conducted after co‐transfecting PPY‐Flag and EGFR‐HA plasmids into CAFs, and the results showed that PPY could bind to EGFR directly (Figure 6J). To verify the role of EGFR in iCAF transformation induced by PPY, we initially conducted PPY knockdown in human CAFs and treated these CAFs with PPY proteins (Figure 6K,L). EGFR knockdown alone exhibited minimal impact on the NF‐κB2 pathway in the absence of stimulants, as compared to the control group (Figure 6L). However, treatment with PPY resulted in a pronounced activation of the NF‐κB2 pathway in the control group, while the EGFR knockdown group exhibited a markedly attenuated response to PPY signals (Figure 6L). In addition, qRT‐PCR and flow cytometry analysis further demonstrated that knocking down EGFR weakened the ability of PPY to induce the iCAF phenotype (**Figure** **7**A–F).

To validate these findings in vivo, we established an orthotopic allograft tumor model by co‐injecting murine pancreatic cancer cells with up‐regulated PPY expression and KPC CAFs with decreased EGFR expression. The IVIS image and gross image demonstrated that mice in the EGFR downregulation group exhibited a significantly smaller tumor volume compared to the control group (Figure 7G,H). Flow cytometry analysis revealed a notable reduction in the proportion of iCAFs, M2 macrophages, and MDSCs within the TME of the EGFR downregulation group (Figure 7I–K). In contrast, there was a higher infiltration of T cells, especially CD8+ T cells, in the TME, accompanied by a decrease in the proportion of exhausted T (Tex) cells (Figure 7L–N).

These results collectively suggest that PPY induces the iCAF phenotype by activating the NF‐κB2 signaling pathway via EGFR. Our findings not only elucidate a novel signaling axis in CAF regulation but also highlight potential therapeutic targets for modulating the PDAC tumor microenvironment. By disrupting the PPY‐EGFR‐NF‐κB2 axis, it may be possible to reprogram the immunosuppressive TME and enhance the efficacy of immunotherapies in PDAC.

### 2.5 PPY Inhibition Enhances PD‐1 Antibody Efficacy in PDAC Treatment

Immunotherapy, particularly PD‐1/PD‐L1 antibodies, has shown remarkable efficacy in various solid tumors.^[^ ^4^ ^]^ Nevertheless, its application in PDAC has encountered significant challenges and disappointing outcomes.^[^ ^4^ ^]^ This predicament is associated with the intricate immunosuppressive tumor microenvironment of PDAC that shields cancer cells from effective cytotoxic immune responses.^[^ ^4^ ^]^ In conjunction with our previous findings, we hypothesized that targeting PPY‐induced iCAFs could potentially enhance the efficacy of PD‐1 antibody treatment by alleviating the immunosuppressive environment.

**Figure 8:**
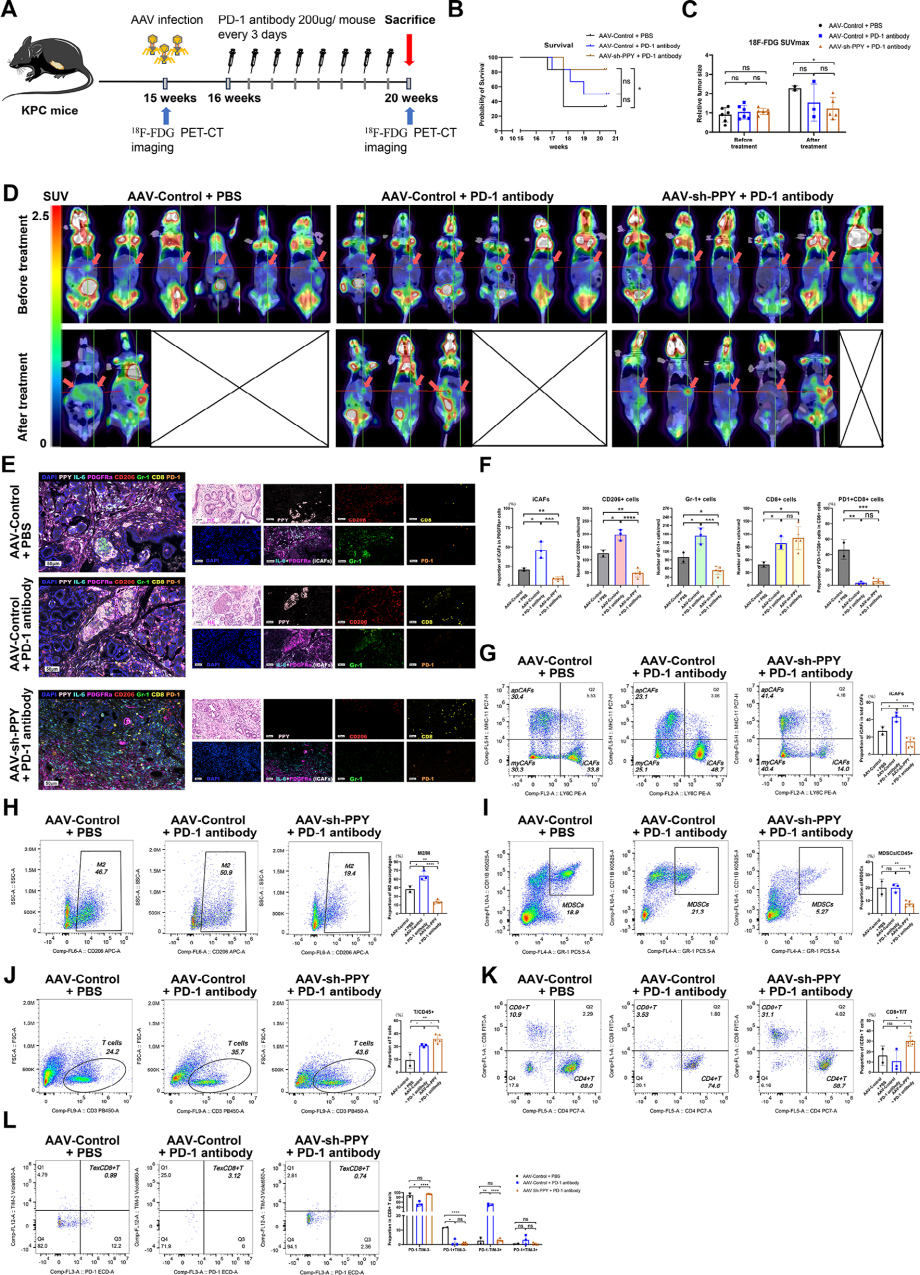
Targeting iCAFs by interfering with PPY enhances the efficacy of anti‐PD‐1 monotherapy in PDAC. A) Schematic diagram illustrating the modeling and drug administration process in KPC mice (n = 6). B) Kaplan‐Meier survival plot depicts overall survival in KPC mice of different groups. *, *p* < 0.05; ns, not statistically significant. C) Summary and statistical analysis of tumor size before and after treatment using ^18^F‐FDG PET‐CT imaging scanning. D) Prior to the treatment, the ^18^F‐FDG PET‐CT imaging system was used to validate tumor formation and size in 15‐week‐old KPC mice. At the terminal stage of the experiments, the same imaging system was utilized again in living KPC mice to estimate tumor volume. E) mIHC staining of PPY (pink), iCAFs (IL‐6, cyan; PDGFRa, magenta), M2 macrophages (CD206, red), MDSCs (Gr‐1, green), CD8^+^T (CD8, yellow), PD‐1^+^cells(orange) in the TME of model mice. Scale bars, 50 µm. F) Summary and statistical analysis of PPY expression and the density of iCAFs and immune cells analyzed by mIHC in the TME of cancer tissues. G–L) Flow cytometric analysis was performed to evaluate the presence of iCAFs (G), M2 macrophages (H), MDSCs (I), T cells (J), CD8+ T cells (K), and exhausted CD8+T cells (L) within the tumor microenvironment of different treatment groups. Statistics are shown in mean ± SD accessed by the unpaired non‐parametric test. *, *p* < 0.05; **, *p* < 0.01; ***, *p* < 0.001; ***, *p* < 0.001; ns, not statistically significant.

To validate our hypothesis, we employed a PD‐1 monoclonal antibody (mAb) and adeno‐associated virus (AAV) carrying sh‐Control/sh‐PPY to treat KPC mice, a PDAC mouse model that closely resembles the occurrence and progression of human PDAC. Before the treatment, ^18^F‐FDG PET‐CT imaging system, a clinical method for early disease detection and treatment response monitoring, was employed to confirm tumor formation and size in 15‐week‐old KPC mice. Subsequently, the mice were randomly divided into three groups based on their relative tumor size (SUVmax): AAV‐Control + PBS group, AAV‐Control + PD‐1 antibody group, and AAV‐sh‐PPY + PD‐1 antibody group. These mice were then administered with AAV containing either sh‐Control or sh‐PPY. Given the time interval required for stable AAV expression, PD‐1 mAb or PBS was administered every 3 days starting from 1 week post‐injection (Figure 1A). The addition of AAV‐sh‐PPY to PD‐1 blockade significantly improved overall survival times compared to the group receiving AAV‐Control and PBS (Figure **8**B). Furthermore, at the study endpoint, the number of surviving mice in the AAV‐sh‐PPY + PD‐1 antibody group exceeded that in both the AAV‐Control + PBS group (5 (83.3%) vs. 2 (33.3%)) and the AAV‐Control + PD‐1 antibody group (5 (83.3%) vs. 3 (50%)) (Figure 8B,D). Additionally, the AAV‐sh‐PPY + PD‐1 antibody group also exhibited a significantly reduced tumor size compared to the AAV‐Control + PBS group (Figure 8C,D). Although no significant difference in tumor volume was observed between the AAV‐Control + PBS group and the AAV‐Control + PD‐1 antibody group, this outcome may be attributed to premature mortality of KPC mice with larger tumors prior to reaching the experimental endpoint (Figure 8C,D). These results highlight the significant role of PPY downregulation in overcoming resistance to anti‐PD‐1 monotherapy in PDAC.

The remaining KPC mice were euthanized, and tumor tissues were collected for further analysis. Subsequent mIHC staining and flow cytometry analysis revealed that mice receiving AAV‐Control and PD‐1 mAb exhibited higher infiltrations of T cells compared to the control group (Figure 8E,F,J). However, this group did not show a statistically significant increase in CD8^+^ T cells, and a higher proportion of these cells expressed the exhaustion marker TIM‐3 (Figure 8K,L). Moreover, the AAV‐Control + PD‐1 antibody group exhibited an increased proportion of iCAF population and higher infiltrations of M2 macrophages and MDSCs compared to the control group (Figure 8E–I). The observed increase in iCAF infiltration along with an exacerbation of the immunosuppressive microenvironment possibly contributed to the limited therapeutic effect of PD‐1 mAb. Notably, when combining PD‐1 antibody treatment with AAV‐sh‐PPY, there was a reduction in iCAF proportion as well as fewer infiltrations of M2 macrophages and MDSCs compared to both the mAb group and control group (Figure 8E–I). Moreover, this combination therapy also led to enhanced infiltration of CD8^+^ T cells within the TME, accompanied by reduced expression of exhaustion markers on these cells (Figure 8K,L).

These results highlight the significant role of PPY downregulation in overcoming resistance to anti‐PD‐1 monotherapy in PDAC. By combining PD‐1 mAb treatment with PPY downregulation, it is possible to enhance the infiltration of functional CD8+ T cells within the TME, thereby inhibiting cancer development and prolonging survival in the PDAC model.

## 3 Discussion

The treatment of PDAC remains a global challenge. Abnormal levels of hormones such as insulin and cholecystokinin have been extensively documented to be closely associated with the occurrence and progression of PDAC.^[^ ^32^, ^33^ ^]^ Recently, it has been reported that elevated levels of PPY lead to increased Ki67 staining in mouse PDAC tissues.^[^ ^35^ ^]^ However, the impact of PPY on the PDAC microenvironment remains unexplored. Recent advances in single‐cell sequencing and spatial transcriptomics have revealed that the PDAC microenvironment is more complex than previously thought, containing extensive CAFs and immunosuppressive cells, particularly at the tumor border.^[^ ^4^, ^5^, ^7^, ^14^, ^48^ ^]^ Our analysis of 82 paired PDAC and adjacent normal tissue samples corroborates these findings (Figure 3H,J). The presence of these cells may contribute to the continuous infiltration and progressive replacement of pancreatic cancer cells into neighboring healthy tissue, emphasizing the need for therapeutic strategies that target the tumor border and enhance effector T cell infiltration into the tumor core.

Through integrated single‐cell RNA sequencing analysis and rigorous experimental validation, our study identifies PPY as a potent inducer of inflammatory CAFs. Notably, PPY‐induced iCAFs exhibit significantly elevated expression levels of key immunomodulatory factors, including IL6, CCL2, and CXCL12. This response pattern distinguishes PPY from previously identified iCAF inducers such as IL‐1, which shows limited impact on CXCL12 expression^[^ ^36^ ^]^ (Figure 3 and Figure S3D–P, Supporting Information). The unique ability of PPY to comprehensively activate the iCAF secretory program suggests its fundamental role in shaping the tumor microenvironment. While abnormal levels of certain hormones have been linked to PDAC progression, the impact of PPY on the tumor microenvironment was previously unknown.^[^ ^32^, ^33^, ^34^ ^]^ In this article, we demonstrated that PPY‐induced iCAFs promote macrophage polarization to an immunosuppressive M2 phenotype and recruit MDSCs, ultimately leading to CD8^+^ T cell exhaustion and an immunosuppressive environment. Furthermore, targeting PPY was found to enhance the therapeutic efficacy of PD‐1 mAb treatment in KPC mice, a well‐established murine model that closely resembles human PDAC.

Our mechanistic investigations employed mRNA sequencing and immunoprecipitation‐mass spectrometry to identify the signaling pathways and receptor interactions mediating PPY‐induced iCAF transformation. mRNA‐seq analysis showed significant upregulation in the expression of RELB, a key signal factor in the NF‐κB2 pathway. In contrast to the classical NF‐κB1 pathway, the NF‐κB2 pathway exhibits a slower but more persistent response,^[^ ^40^ ^]^ which is consistent with our previous experimental findings that the expression alterations in iCAF markers manifested ≈12 h after PPY treatment. Western blot analysis revealed that PPY treatment upregulated the expression of NF‐κB2 markers. Conversely, when cancer cells with reduced PPY expression were co‐incubated with CAFs, the expression of NF‐κB2 markers in CAFs was downregulated. Further investigation using IP‐MS and Co‐IP, along with EGFR knockdown experiments, indicated that PPY may activate NF‐κB2 to promote the transformation of iCAFs primarily by interacting with EGFR on the surface of CAFs. Recent research has revealed complex roles for EGFR in CAF regulation. While TGF‐β‐induced EGFR expression in murine pancreatic stellate cells (PSCs) was reported to promote myCAF characteristics, EGFR knockout was found to downregulate both myCAF and iCAF signatures in PDAC organoid co‐cultures.^[^ ^30^ ^]^ This phenomenon can be attributed to the intricate structure and function of EGFR, which consists of four regions in the extracellular binding domain that bind different ligands and transduce diverse signals.^[^ ^49^ ^]^ The EGFR phosphorylation site reported in this article is Y1068, which plays a crucial role in activating the MAPK pathway by recruiting and activating GRB2. However, our mRNA‐seq and experimental results indicated that GRB2 was downregulated, whereas PLCγ, which is recruited and activated by phosphorylation at Y992, was significantly upregulated. This selective pathway activation distinguishes PPY's effects from those of TGF‐β, which operates through Y1068 phosphorylation. PLCγ serves as an essential signal transduction molecule in various immune pathways, including the NF‐κB pathway.^[^ ^50^, ^51^, ^52^ ^]^ In addition, the Western Blot results and bioinformatics analysis showed that PPY has little effect on EGFR expression but significantly promotes the phosphorylation of EGFR. Additionally, a phase III clinical trial has reported that targeting EGFR prolongs the survival time of PDAC patients.^[^ ^47^ ^]^ Therefore, it is imperative to comprehensively elucidate the structure and functions of EGFR to optimize PDAC treatment.

apCAF has recently been identified as a contributor to the immunosuppressive microenvironment in PDAC by promoting the expansion of regulatory T cells.^[^ ^53^ ^]^ However, we did not observe a significant correlation between the proportion of apCAFs and PPY‐induced iCAFs in both in vivo and in vitro experiments (Figure 2). This may be attributed to differences in their origins: apCAFs derive from mesothelial cells whereas iCAF and myCAF originate from resident fibroblasts within the original tissue.^[^ ^2^, ^14^, ^53^ ^]^ This makes mutual transformation more likely to occur between iCAFs and myCAFs, while regulation of apCAF proportion might involve other components or molecules present in TME.^[^ ^2^, ^14^, ^53^ ^]^ Considering the complexity and plasticity of TME, further investigations are warranted to elucidate the underlying mechanisms that drive phenotypic transformation and functions of specific cancer‐promoting CAF populations, thereby providing a basis for developing targeted treatment strategies.

In conclusion, our study screened and validated PPY as a potent inducer of iCAFs in vitro and in vivo, and elucidated its mechanism of action through the PPY‐EGFR‐NF‐κB2 axis. The crucial role of PPY‐induced iCAFs in creating an immunosuppressive microenvironment in PDAC highlights its potential as a therapeutic target to enhance immunotherapy efficacy. This work provides a foundation for developing combination therapies that target the communication between pancreatic cancer cells and CAFs, potentially improving outcomes for PDAC patients.

## 4 Experimental Section

### Single‑Cell RNA‑Sequencing Data Analysis

The scRNA‐seq data of pancreatic cancer was obtained from the Gene Expression Omnibus (GEO) database (accession numbers: GSE111672, GSE154778, GSE155698, and GSE156405) and Genome Sequence Archive (GSA) database (accession numbers: CRA001160), while scRNA‐seq data of normal pancreas was acquired from GEO database (accession numbers GSE165399, GSE81547, and E‐MTAB‐5061). The Harmony algorithm was used for batch integration and correction. Only cells with at least 500 genes and no more than 15% of the total mitochondrial feature count were retained for the analysis. Normalization was performed using the log‐normalization method. Clustering was performed using 15 principal component analyses with a resolution parameter of 0.1. The patients were stratified into two groups based on iCAF abundance and proportion: those in the top quartile were classified as the high iCAF group, while the remaining patients were assigned to the low iCAF group. The differentially expressed genes (DEGs) in each cluster were identified using the FindAllMarkers function in Seurat, and a heat map was displayed and showed that the differences of each group were obvious. Cell types for each cluster were annotated using the CellMarker 2.0 database (http://bio‐bigdata.hrbmu.edu.cn/CellMarker/). The 2D visualization of the clusters was performed with t‐distributed Stochastic Neighbor Embedding (tSNE) and uniform manifold approximation and projection (UMAP). The differences in pathway activities of each cluster were scored by Gene Set Variation Analysis (GSVA), and the copy number variation (CNV) was evaluated using inferCNVpy package. The DEGs in the cancer cells were analyzed using DEsingle algorithm, and they were intercrossed with secreted protein genes downloaded by the Human Protein Atlas (HPA) database.

### Patient Specimens

The 10 surgery samples for CAF isolation and subsequent experiments were obtained from patients with PDAC who underwent curative resection at the Tongji Hospital, Huazhong University of Science and Technology (HUST, Wuhan, China). All related experiments were approved by the Medical Ethics Committee of Tongji Medical College, Huazhong University of Science and Technology (TJ‐IRB202405028), and informed consent was obtained from all subjects. The PDAC tissue microarrays (HPanA170Su05) were purchased from Shanghai Outdo Biotech Company (Shanghai, China), which was approved by China Human Genetic Resources Management Office.

### Mice and Tumor Models

All the animal experiments were approved by The Laboratory Animal Welfare and Ethics Committee of Tongji Hospital, Huazhong University of Science and Technology (TJ‐202212046). 7‐week‐old male C57BL/6J mice were obtained from Beijing Vital River Laboratory Animal Technology Co., Ltd. All the mice were housed in the Laboratory Animal Center of Tongji Hospital under specific pathogen‐free conditions. All animal experiments were randomized and conducted in accordance with the Guide for the Care and Use of Laboratory Animals. In the orthotopic allograft model of mice, 1 × 10^6^ Panc02 cells regulated PPY expression and labeled with luciferase were combined with 2.5 × 10^5^ KPC CAFs and orthotopically injected into the body and tail of the mouse pancreas. 3 weeks after tumor modeling, the tumor volume was quantified using an IVIS imaging system following intraperitoneal administration of 200 µl of D‐Luciferin potassium (15 mg mL^−1^; HY‐12591, MCE) dissolved in PBS. The mice were subsequently euthanized, and the cancer tissues were then isolated for subsequent experimental procedures. The parental mice of KPC (*Kras* ^LSL‐G12D/+^; *Trp53* ^LSL‐R172H/+^; *Pdx1*‐Cre) mice were introduced from Shanghai Model Organisms Center, Inc., and bred in the Laboratory Animal Center of Tongji Hospital under specific pathogen‐free conditions. Before formal experiments, the genotype of all KPC animals was confirmed as described previously.^[^ ^8^ ^]^ The 18F‐FDG PET‐CT imaging system was used to validate tumor formation and size in 15‐week‐old KPC mice, and the mice were randomly divided into three groups based on their relative tumor size (SUVmax). These mice were then administered with AAV‐PAN (5 × 10^11^ mouse^−1^) containing either sh‐Control or sh‐PPY, PD‐1 mAb (200 µg mouse^−1^ dissolved in PBS) or PBS is administered every 3 days starting from 1 week post‐injection.

### Cell Lines and Primary Cells

Human pancreatic cancer cell lines AsPC‐1 (RRID: CVCL_0152), BxPC‐3 (RRID: CVCL_0186), SW1990 (RRID: CVCL_1723), Panc 08.13 (RRID: CVCL_1638), HPAF‐II (RRID: CVCL_0313), Capan‐2 (RRID: CVCL_0026) were purchased from the American Type Culture Collection (ATCC, Manassas, VA, USA). Human pancreatic cancer cell line PANC‐1 (RRID: CVCL_0480), human ductal cell hTERT‐HPNE (RRID: CVCL_C466), and human embryonic kidney cell line HEK 293T (RRID: CVCL_0063) were obtained from the Cell Resource Center, Institute of Biochemistry and Cell Biology at the Chinese Academy of Science (Shanghai, China). Mouse pancreatic cancer cell line Panc02 (RRID: CVCL_D627) was acquired from Boster Biological Technology (Wuhan, China) and cultured in DMEM supplemented with 10% fetal bovine serum (FBS) at 37 °C and 5% CO2. THP‐1 cells (RRID: CVCL_0006) were a kind gift from Hepatic Surgery Center of Tongji Hospital, and cultured in RPMI‐1640 with 10%FBS, 0.05 mM β‐mercaptoethanol, and 1%P/S. All cell lines used for in vitro and in vivo experiments were within ten passages. CAFs were isolated from fresh pancreatic cancer tissues of patients with PDAC or KPC mice as described previously,^[^ ^8^ ^]^ and cultured in DMEM supplemented with 10% FBS and 1% penicillin‐streptomycin (PS), and they were utilized for experiments within eight passages. Primary bone marrow cells were isolated from the bilateral femurs and fibulae of wild‐type C57BL/6J mice, and cultured in RPMI‐1640 supplemented with 10% FBS and 1% PS, awaiting further treatment. All primary cells used for in vitro and in vivo experiments were within seven passages.

### RNA Extraction and qRT‐PCR

The human or mouse CAFs were collected following treatment with the corresponding recombinant proteins (Human PPY protein (HY‐P71062, MCE), Human IL‐1α protein (HY‐P7027, MCE), Mouse PPY protein (RPB265Mu01, Cloud‐Clone Corp.), Mouse IL‐1α protein (HY‐P7072, MCE)) or supernatants for the designated durations. Total RNA was extracted using the TRlzol reagent (9108, Takara), followed by reverse transcription using a PrimeScript FAST RT reagent Kit with gDNA Eraser (RR092S, Takara). Real‐time quantitative PCR was performed in triplicate, utilizing the ChamQ Universal SYBR qPCR Master Mix (Q711‐02, Vazyme). The primers (Table S1, Supporting Information) were synthesized by Sangon Biotech (Shanghai) Co., Ltd.

### Western Blot

Western blotting analysis was performed as described previously (5). The following antibodies were used for Western blotting: anti‐ACTA2 (BM0002, BOSTER), anti‐Beta‐Actin (66009‐1‐Ig, Proteintech), anti‐GAPDH (60004‐1‐Ig, Proteintech), anti‐RELB (A23389, ABclonal), anti‐NF‐KB2 (HY‐P80763, MCE), anti‐Phos‐NF‐KB2 (AP1391, ABclonal), anti‐EGFR (SP00‐86, HUABIO), anti‐Histone (BM4148, BOSTER), anti‐ Phos‐EGFR (AP0026, ABclonal), anti‐PLCG2(A25017, ABclonal), anti‐phos‐PLCG2(AP1519, ABclonal), anti‐phos‐PLCG2 (A19059, ABclonal).

### Plasmid Construction, Virus Packaging, and Infection

The target gene sequences were downloaded from the NCBI database (https://www.ncbi.nlm.nih.gov/), and the primers were designed (Table S2, Supporting Information) and then synthesized by Sangon Biotech (Shanghai) Co., Ltd. The target gene sequences were amplified using 2 × Phanta Flash Master Mix (P510‐01, Vazyme) with cDNA derived from PDAC tissues and cell lines serving as PCR templates. Following cleavage of the fragment and vector with restriction enzymes (Thermo Fisher), the sequences were respectively constructed in vectors using ligation high (LGK‐100, TOYOBO). The authenticity of the plasmid and its insertion sequences were confirmed by Sanger sequencing from Sangon Biotech (Shanghai) Co., Ltd. Plasmids containing target genes or sequences, along with a lentiviral vector packaging system, were co‐transfected into HEK 293T cells using Polyethylenimine Linear (PEI) MW40000 (40816ES02, YEASEN). 72 h post‐transfection, the supernatants were harvested and filtered through 0.22 µm filters (SLGP033RB, Millipore). Subsequently, these supernatants were employed for cell infection facilitated by Plybrene (40804ES76, Yeasen). After 12 h, the viral supernatant was replaced with fresh cell culture mediums, and puromycin (BL528A, Biosharp) was administered to eliminate untransfected cells after 24 h.

### ELISA

The cells of each group were uniformly and equally seeded in the culture dish and treated for the designated duration. The supernatant was collected and combined spun down, and assayed as the manufacturer's protocol. ELISA assays used were Mouse CXCL12 ELISA kit (KE10049, Proteintech), Mouse IL‐6 ELISA kit (EK206HS, MULTI SCIENCES), Mouse CCL2 ELISA kit (EK287, MULTI SCIENCES), Human CXCL12 ELISA kit (EK1119, MULTI SCIENCES), Human CCL2 ELISA kit (EK187, MULTI SCIENCES), and Human IL‐6 ELISA kit (EK106, MULTI SCIENCES).

### Macrophage Polarization

Primary bone marrow cells were isolated from the bilateral femurs and fibulae of wild‐type C57BL/6J mice. The freshly isolated marrow cells were treated with 10 ng mL^−1^ M‐CSF (HY‐P7085, MCE) for a duration of 7 days to induce their differentiation into primary mouse macrophages. The THP‐1 cells were differentiated into macrophages by treating them with 100 ng mL^−1^ PMA (HY‐18739, MCE) for 3 days prior to the relative experiments. After sorting and purifying these stimulated cells using flow cytometry, they were utilized in subsequent experiments. The murine or human macrophages were cocultured with indicated iCAFs using a 0.4 µm Transwell system (3412, CORNING) or treated with iCAF supermants for 3 days. Macrophages were subsequently for mRNA isolation or flow cytometry analysis. For the flow cytometry analysis of macrophage polarization, anti‐mouse CD45‐APC/Cy7 (557659, BD), CD11b‐BV510 (562950, BD), F4/80‐PE (565410, BD), and CD206‐Alexa Fluor 647 (565250, BD) are used to stain murine macrophages, while anti‐human CD45‐APC/Cy7 (557833, BD), CD11b‐BV510 (562950, BD), CD68‐Alexa Fluor 647 (562111, BD), HLA‐PE/Cy7 (560651, BD), and CD206‐PE (555954, BD) are utilized for human macrophages.

### MDSC Migration Assay

MDSCs were generated by culturing the primary bone marrow cells in RPMI‐1640 medium containing 10 ng mL^−1^ GM‐CSF (HY‐P7361, MCE) for 4 days. Cell purity was determined by flow cytometric analysis using anti‐CD11b and Gr‐1 antibodies (>95%), and cell viability was checked by Trypan blue dye exclusion. 2 × 10^5^ MDSCs in 150 µl of serum‐free medium were seeded in the upper chamber of the 5 µm Transwell system (3421, CORNING), while 650 µl of iCAF supernatants were added to the receiving chamber to facilitate cell migration for a duration of 4 h. Following migration, the cells that had migrated to the basal side of the inserts were fixed in 4% polyformaldehyde for 5 h and stained with 2% Crystal Violet.

### Flow Cytometry

For the flow cytometry analysis performed on the in vitro experimental cells, the treated cells were pre‐incubated with CD16/32 (65057‐1‐Ig, Proteintech) and subsequently subjected to viability staining (564997, BD). For flow cytometric analysis of myCAF, iCAF, and apCAF populations, cells were stained for 30 min with anti‐mouse CD31‐FITC (102405, BioLegend), CD45‐APC/Cy7 (557659, BD), CD326 (Ep‐CAM)‐BV421 (118225, BioLegend), PDPN‐APC (127410, BioLegend), Ly6C‐PE (128008, BioLegend), and I‐A/I‐E (MHC‐II)‐PE/Cy7 (107630, BioLegend). After fixation with paraformaldehyde, the cells were subjected to flow cytometric detection and analysis (Beckman CytoFLEX). PDPN^+^CD45^−^EPCAM^−^ cells were identified as CAFs, which were further classified into iCAF (Ly6C^+^MHC‐II^−^), myCAF (Ly6C^+^MHC‐II^−^), and apCAF (Ly6C^+^MHC‐II^−^) populations.

For the flow cytometry analysis performed on cancer tissues, the freshly dissected tumor tissues were dissociated into single cells using a Tumor Dissociation Kit (130‐096‐730, Miltenyi), gentleMACS C Tubes (130‐093‐237, Miltenyi), and gentleMACS Dissociator Instruments. After cell counting, the cells were then added to their respective flow tubes for CD16/32 (65057‐1‐Ig, Proteintech) blocking and viability staining (564997, BD). The histological staining protocol for CAFs was identical to that used in vitro cytology. The staining scheme utilized for immune cells in the cancer tissues was as follows: CD45‐APC/Cy7 (557659, BD), CD11b‐BV510 (562950, BD), F4/80‐PE (565410, BD), CD206‐Alexa Fluor 647 (565250, BD), Gr‐1‐ PerCP/Cy5.5 (552093, BD), CD3e‐BV421 (562600, BD), CD4‐PE/Cy7 (552775, BD), PD‐1‐PE‐CF594 (562523, BD), TIM‐3‐BV650 (747623, BD).

### mIHC Staining and Analysis

The PDAC tissue microarray (HPanA170Su05) purchased from Shanghai Outdo Biotech Company and wax blocks of experimental mouse tissue were sent to Wuhan Human Genetic Resource Bank (Wuhan Biobank Co., LTD) for mIHC staining and analysis. The tissue sections were subjected to deparaffinization, followed by endogenous peroxidase blocking and heat‐induced antigen retrieval. Subsequently, the IHC process was performed through sequential multiple iterative cycles. The antibody panel for the PDAC tissue microarray were as follows: PPY (15493‐1‐AP, Proteintech, 1:1000), IL‐6 (A21264, Abclonal, 1:1000), PDGFRa (ET1702‐49, HAUBIO, 2:100), CD206 (24595, CST, 1:1500), CD33 (ab269456, Abcam, 1:50), CD8 (85336, CST, 1:400), PD‐1 (43248, CST, 1:200). The antibody panel for the cancer tissues of mice models was as follows: PPY (15493‐1‐AP, Proteintech, 1:1000), IL‐6 (YA3428, MCE, 1:1000), PDGFRa (ET1702‐49, HAUBIO, 2:100), CD206 (24595, CST, 1:1500), Gr‐1 (14‐5931‐82, Thermo Fisher, 1:50), CD8 (98941, CST, 1:1000), PD‐1 (66220‐1‐Ig, Proteintech, 1:2000). Multiple iterative cycles of the immunohistochemistry (IHC) process were conducted, involving staining, scanning, and chromogen stripping. The antibodies and Opal Polaris 7 Color IHC DETECTION KIT (NEL871001KT, AKOYA) were utilized in this procedure. Fluorescence images were acquired using PhenoImager HT System equipped with inForm2.6 software. For phenotype and quantitation analysis, a nuclear segmentation algorithm was utilized to accurately identify individual cells based on the DAPI image. Subsequently, location information and expression levels of all markers were computed for each identified cell.

### mRNA Sequencing and Analysis

CAFs from 3 PDAC patients were treated with PPY recombinant proteins (40 ng mL^−1^) for 24 h, and the CAFs were subsequently collected and treated with TRlzol reagent (9108, Takara). Then samples were sent to the Sangon Biotech (Shanghai) Co., Ltd. for library preparation and sequencing. The RNA sequencing was performed using the Illumina Hiseq2000 Genome Analyzer instrument (Illumina, San Diego, CA), and the RNA‐seq data has been submitted to the NCBI Sequence Read Archive (SRA) database with the accession ID PRJNA1160433. FastQC (version 0.11.2) was utilized for assessing the quality of sequenced data and eliminating low‐quality bases. Data were then mapped to the reference genome by HISAT2 (version 2.0), and differentiated gene expression was analyzed using DESeq2 (version 1.12.4). The differentially expressed genes were subjected to KEGG and GO functional enrichment analyses to identify the associated enriched pathways.

### IP‐MS and Co‐IP

The gene sequence of EGFR and the predominant PPY splicing variant was downloaded from the NCBI database (https://www.ncbi.nlm.nih.gov/), and the primers were designed (Table S2, Supporting Information) and subsequently synthesized by Sangon Biotech (Shanghai) Co., Ltd. The N‐terminus of the sequences of EGFR and PPY in the overexpression plasmids are tagged with HA and Flag, respectively. The supernatant of PPY‐Flag was collected from HEK 293T cells transfected with the plasmids for 3 days and concentrated using ultrafiltration tubes (UFC900396, Millipore). Human CAFs are treated with enriched PPY‐Flag supernatant for 24 h, and these cells were then collected and subject to immunoprecipitation using an anti‐Flag antibody (66008‐4‐Ig, Proteintech) or IgG antibody (30000‐0‐AP, Proteintech). The pull‐down proteins were subsequently sent to BGI Genomics Co., Ltd. for mass spectrometric detection using the Q‐Exactive HF X mass spectrometer. A total of 1488 proteins in the IP group and 1577 proteins in the IgG group were successfully identified and matched. For the Co‐IP assay, the enriched PPY‐Flag supernatant was utilized to treat human CAFs which were transfected EGFR‐HA overexpression plasmids for 24 h. Subsequently, these cells were collected, and the anti‐Flag antibody (66008‐4‐Ig, Proteintech) was used to pull down PPY and proteins bound to it. Western blotting was then performed using an anti‐Flag antibody (66008‐4‐Ig, Proteintech) and an anti‐HA antibody (81290‐1‐RR, Proteintech).

### Molecular Docking

To understand the interaction of PPY with the extracellular domain of EGFR, molecular docking was conducted using HDOCK server (http://hdock.phys.hust.edu.cn/). The tertiary structures of PPY (AlphaFold: AF‐P01298‐F1) and EGFR (PDB: 7SYD) were obtained from Uniprot (https://www.uniprot.org/). The best model was selected based on docking scores from the top ten models, and the resulting docked complexes were visualized using PyMOL. Subsequently, Ligplot + v2.2 software was used to analyze the interaction between the receptor and ligand along with its 2D representation.

### ^18^F‐FDG PET‐CT Imaging

The KPC mice were fasted for 12 h prior to the PET‐CT scan, and ^18^F‐FDG was injected via the tail vein 1 h before imaging. The mice were anesthetized with isoflurane, and the PET‐CT imaging was conducted using U‐EXPLORER PET/CT. The IRIS PET/CT software was utilized for quantifying the ^18^F‐FDG uptake in the tumor tissue, with measurement of the maximum uptake value (SUVmax).

### Statistical Analysis and Reproducibility

Statistical analyses and data plotting were performed in the R (v4.4.0), GraphPad Prism 9.0 software, and FlowJo (version 10.4, BD Life Sciences). For the data approximately normally distributed, Student's *t‐*test were performed. Survival analysis was conducted using a log‐rank test. *p* < 0.05 was considered statistically significant for each comparison.

### Data Availability

The scRNA‐seq data of pancreatic cancer was obtained from the Gene Expression Omnibus (GEO) database (accession numbers: GSE111672, GSE154778, GSE155698, and GSE1556405) and Genome Sequence Archive (GSA) database (accession numbers: CRA001160), while scRNA‐seq data of normal pancreas was acquired from GEO database (accession numbers: GSE165399, GSE81547) and ArrayExpress database (accession number: E‐MTAB‐5061). The bulk RNAseq data we performed on CAFs treated with PPY, as well as the control CAFs, has been deposited in the NCBI SRA database under the accession ID PRJNA1160433. All other raw data can be provided upon request from the corresponding author.

## Conflict of Interest

The authors declare no conflict of interest.

## Author Contributions

M.C., B.C., and Y.Z. conceived and designed the study. M.C., W.P., L. L., and H.H. performed the experiments. L.L. performed PET‐CT imaging scans and analysis. M.C. analyzed the data and wrote the paper. R.W., W.C., Y. Z., S.C., H.C., J.L., S.X., S.B. and Q.Z. revised the paper. All authors read and approved the final manuscript.

## Supporting information



Supporting Information

Supplemental Table 1

Supplemental Table 2

## Data Availability

The data that support the findings of this study are openly available in The NCBI SRA database at https://www.ncbi.nlm.nih.gov, reference number 1160433.
