# Supplementary material for: PPY‐Induced iCAFs Cultivate an Immunosuppressive Microenvironment in Pancreatic Cancer
Source: Adv Sci (Weinh). 2025 Mar 31;12(20):2413432. doi: 10.1002/advs.202413432 (PMC12120788; doi:10.1002/advs.202413432)
Supplement: Supplementary file 1 — Supporting Information [file ADVS-12-2413432-s001.docx]

**
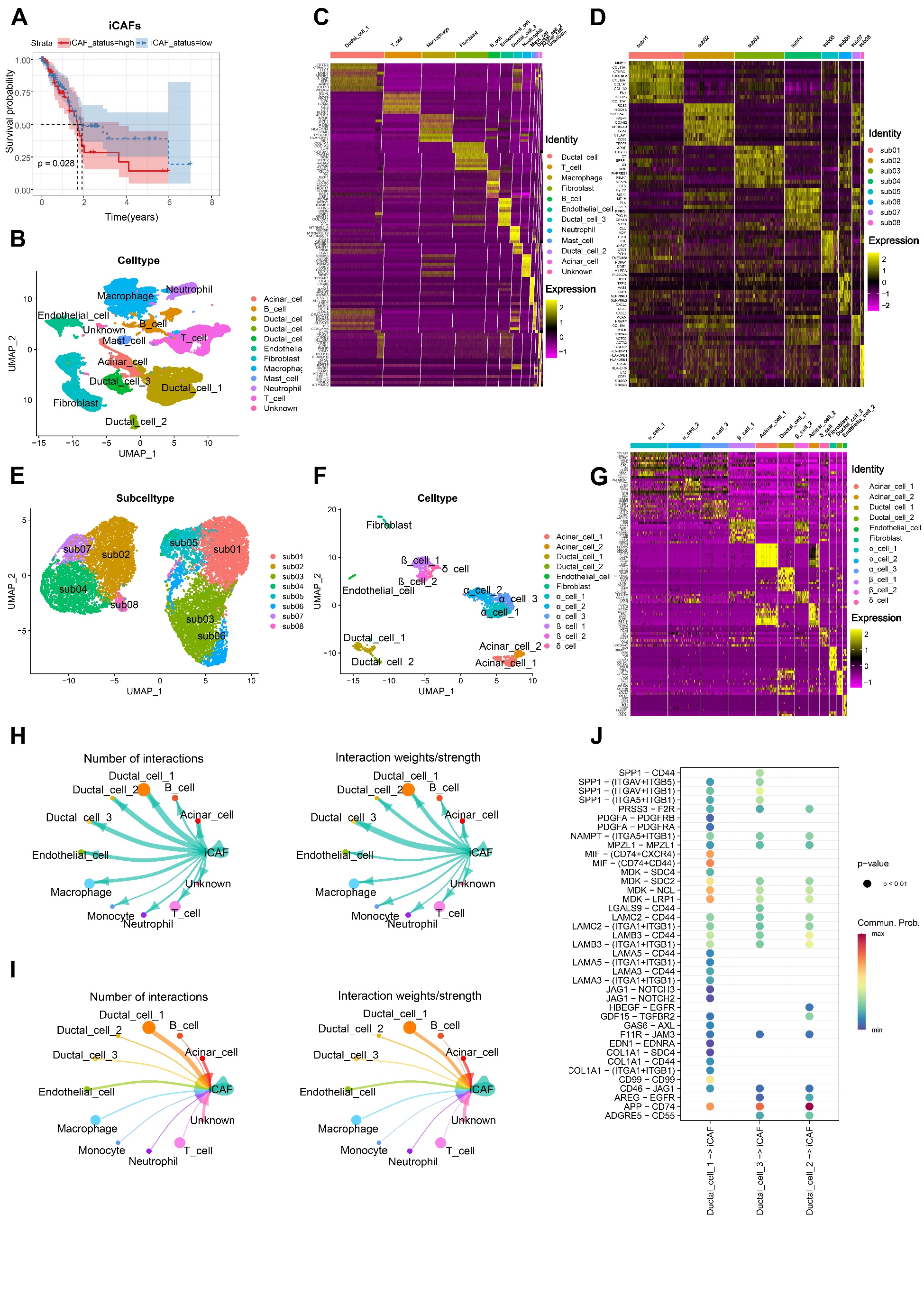
**

**Supplementary Figure S1. Screening and initial validation candidates capable of significantly inducing iCAF phenotype.** **A,** The correlation between iCAFs abundance and overall survival times was analyzed using TCGA expression and survival data. **B,** The Uniform Manifold Approximation and Projection (UMAP) plot of the 92,222 cells in the single-cell sequencing profile revealed distinct cell types observed in PDAC. **C,** The heatmap showing expression levels of specific markers in each cell type in PDAC. **D,** The heatmap exhibiting different expression profiles in each subtype of fibroblasts in PDAC. **E,** The UMAP plot exhibited diverse subtypes of fibroblasts observed in PDAC. **F,** The UMAP plot of the 1,887 cells in the single-cell sequencing profile revealed distinct cell types observed in normal pancreas. **G,** The heatmap showed the expression levels of specific markers in each cell type in the normal pancreas. H and I, CellChat analysis was conducted to evaluate the number and strength of interactions from iCAFs to other cell types in the scRNA-seq dataset (H), as well as the interactions from other cell types in the scRNA-seq dataset to iCAFs (I). J, Bubble plots illustrate the highest-ranked communication pairs from three populations of ductal cells of PDAC to iCAFs.





**Supplementary Figure S2. Screening and initial validation of cancer cell-secreted proteins associated with iCAF induction. A,** The t-SNE plot displayed the origin of PDAC fibroblasts, with each cell color-coded according to the corresponding patient information. **B and C,** The copy numbers of cells were analyzed by inferCNVpy, and the heatmap (B) and t-SNE plot (C) were used to show the copy number variation (CNV) in cell clusters. **D-F,** The Kaplan Meier Plotter database was used to assess the correlation between the expression of PRAP1 (D), PPY (E), and APOE (F) with the overall survival of PDAC patients. **G,** The TCGA expression and surviving data were used to analyze the correlation between the expression of LY96 with the overall survival of PDAC patients. **H-K,** The Kaplan Meier Plotter database was used to assess the correlation between the expression of TREM2 (H), DEFB1(I), EDN2 (J), and ISG15 (K) with the overall survival of PDAC patients.

**
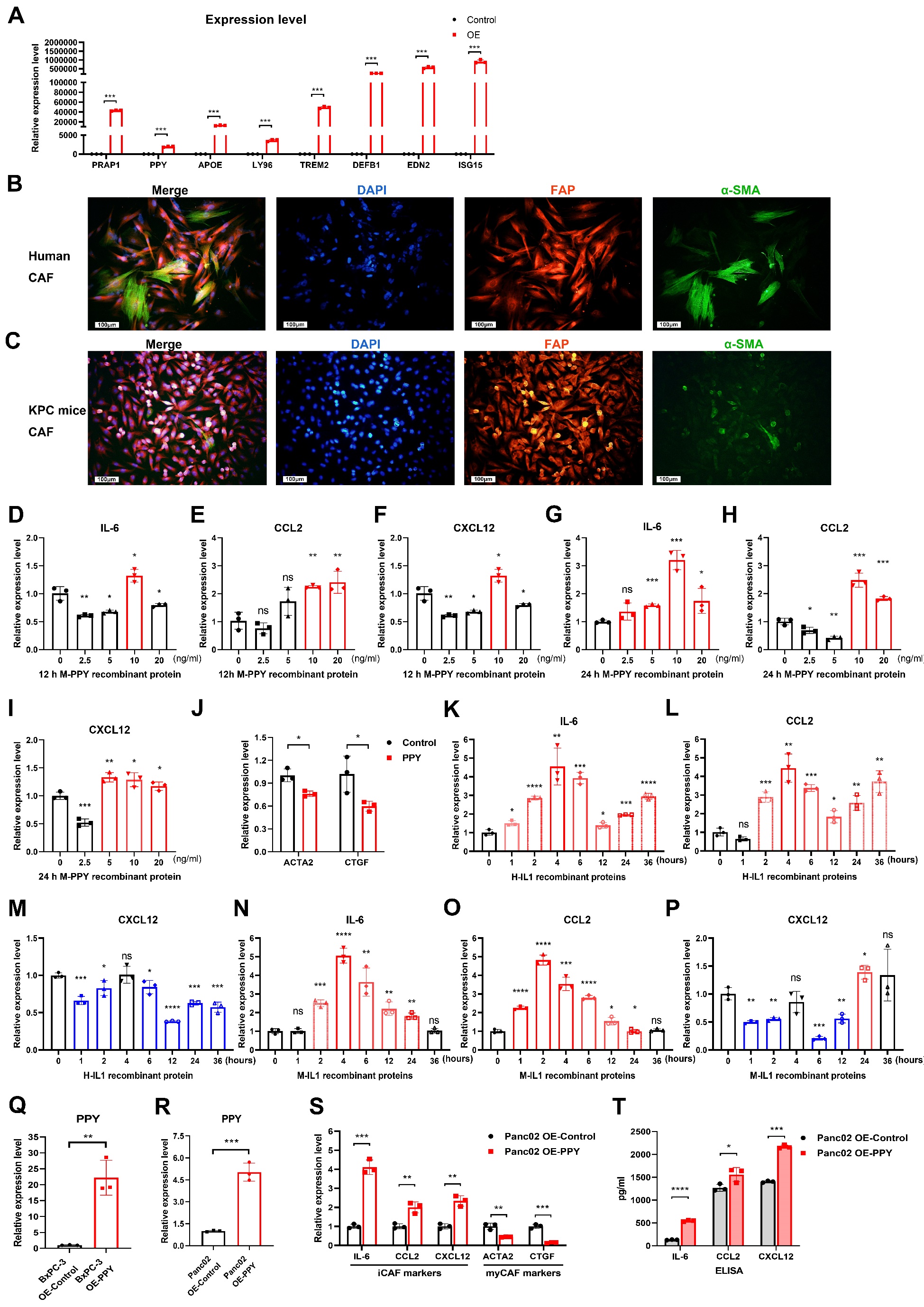
**

**Supplementary Figure S3. The iCAF phenotype in PDAC CAFs is significantly induced by PPY both in vitro and in vivo.** **A,** The plasmids expressing the candidates were separately constructed and transfected into HEK 293T cells, followed by quantification of the upregulation efficiency using qRT-PCR. **B and C,** The CAFs isolated from cancer tissues of PDAC patients (B) and KPC mice (C) were subjected to immunostaining to examine their morphology as well as the expression levels of FAP (red) and α-SMA (Green). Scale bars, 100 μm. **D-F,** After treating murine CAFs with various concentrations of PPY protein for 12 hours, qRT-PCR analysis was performed to assess their alterations in the expression levels of iCAF markers (IL-6 (D), CCL2 (E), and CXCL12 (F)). **G-I,** After treating murine CAFs with various concentrations of PPY protein for 24 hours, qRT-PCR analysis was performed to assess their alterations in the expression levels of iCAF markers (IL-6 (G), CCL2 (H), and CXCL12 (I)). **J,** qRT-PCR analysis of the expression levels of myCAF markers (ACTA2 and CTGF) after treating the murine CAFs with PPY proteins (10ng/ml) for 24 hours. **K-M,** PCR analysis showed the levels of IL-6 (K), CCL2 (L), and CXCL12 (M) in human CAFs treated with human IL1α (1 ng/ml) at different time points. **N-P,** PCR analysis showed the levels of IL-6 (N), CCL2 (O), and CXCL12 (P) in KPC CAFs treated with murine IL1α (10 ng/ml) at different time points. Each experiment was performed three times independently, and Student’s t-test was used to analyze the data. The results are presented as mean ± SD; *, *P* < 0.05; **, *P* < 0.01; ***, *P* < 0.001; ***, *P* < 0.001; ns, not statistically significant. OE, overexpression.


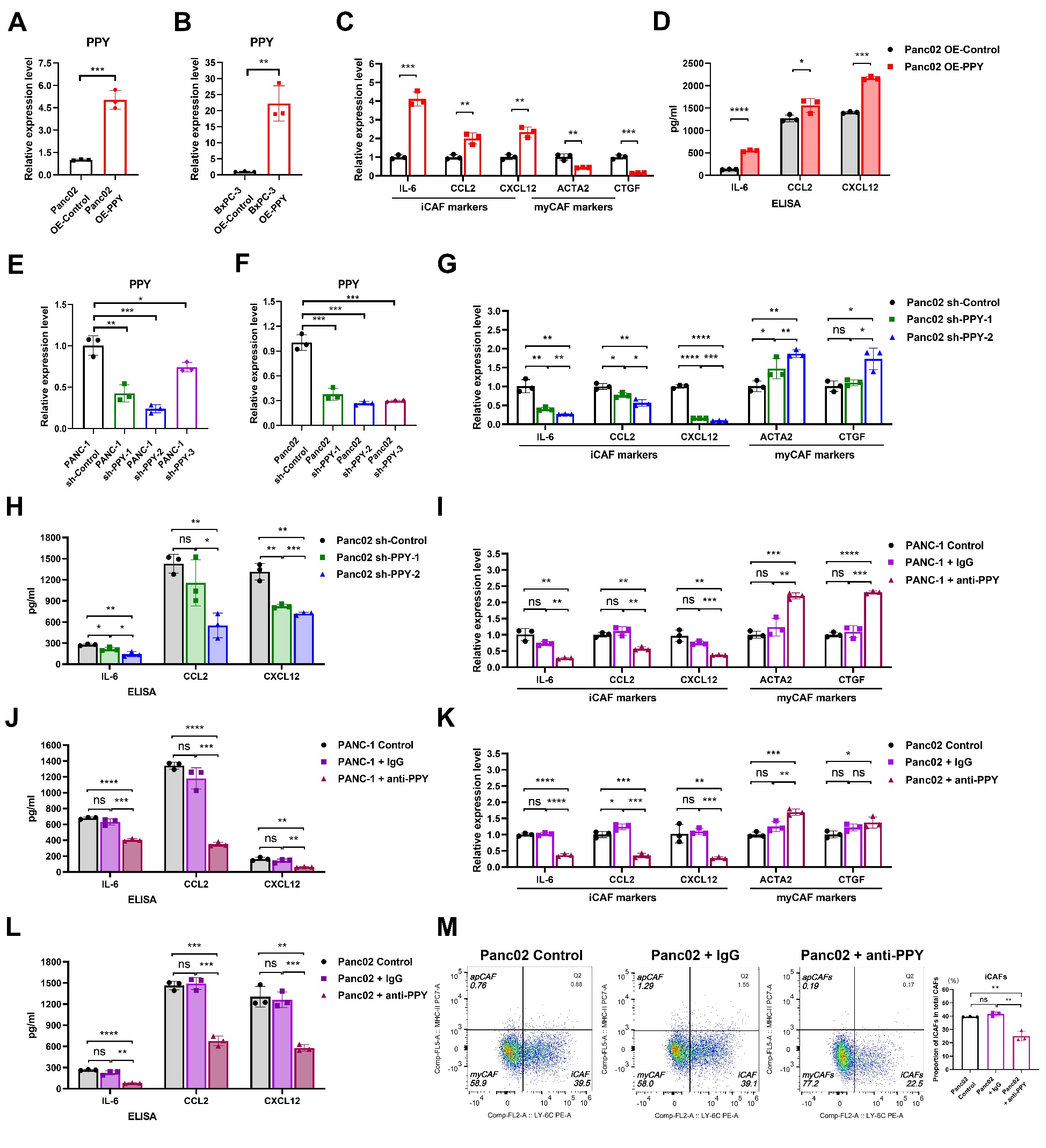


**Supplementary Figure S4. The promotion effects of cancer cells on the iCAF phenotype were attenuated upon targeting PPY.** **A and B,** The efficiency of up-regulation of PPY expression in BxPC-3 cells (A) and Panc02 cells (B) was assessed by qRT-PCR. **C and D,** After co-culturing murine CAFs with Panc02 cells that had up-regulated PPY expression for 24 hours, the expression levels of iCAF markers (IL-6, CCL2, and CXCL12) and myCAF markers (ACTA2 and CTGF) were analyzed by qRT-PCR (C), and secretion levels of IL-6, CCL2, and CXCL12 were assessed by ELISA (D). **E and F,** The efficiency of down-regulation of PPY expression in PANC-1 cells (E) and Panc02 cells (F) was assessed by qRT-PCR. **G and H,** After co-culturing murine CAFs with Panc02 cells that had down-regulated PPY expression for 24 hours, the expression levels of iCAF markers (IL-6, CCL2, and CXCL12) and myCAF markers (ACTA2 and CTGF) were analyzed by qRT-PCR (G), and secretion levels of IL-6, CCL2, and CXCL12 were assessed by ELISA (H). **I and J,** After adding the PPY antibody to the co-culture system of human CAFs and cancer cells (PANC-1) for 24 hours, the expression levels of iCAF markers (IL-6, CCL2, and CXCL12) and myCAF markers (ACTA2 and CTGF) were analyzed by qRT-PCR (I), and secretion levels of IL-6, CCL2, and CXCL12 were assessed by ELISA (J). **K and L,** After adding the PPY antibody to the co-culture system of human CAFs and cancer cells (PANC-1) for 24 hours, the expression levels of iCAF markers (IL-6, CCL2, and CXCL12) and myCAF markers (ACTA2 and CTGF) were analyzed by qRT-PCR (K), and secretion levels of IL-6, CCL2, and CXCL12 were assessed by ELISA (L). **M,** After adding PPY antibody to the co-culture system of KPC mice-derived CAFs and cancer cells (Panc02) for 24 hours, flow cytometry analysis was performed to evaluate the populations of iCAFs (Ly6C+MHC-II-), myCAFs (Ly6C+MHC-II-), and apCAFs (Ly6C+MHC-II-). Each experiment was performed three times independently, and Student’s t-test was used to analyze the data. The results are presented as mean ± SD; *, *P* < 0.05; **, *P* < 0.01; ***, *P* < 0.001; ***, *P* < 0.001; ns, not statistically significant.


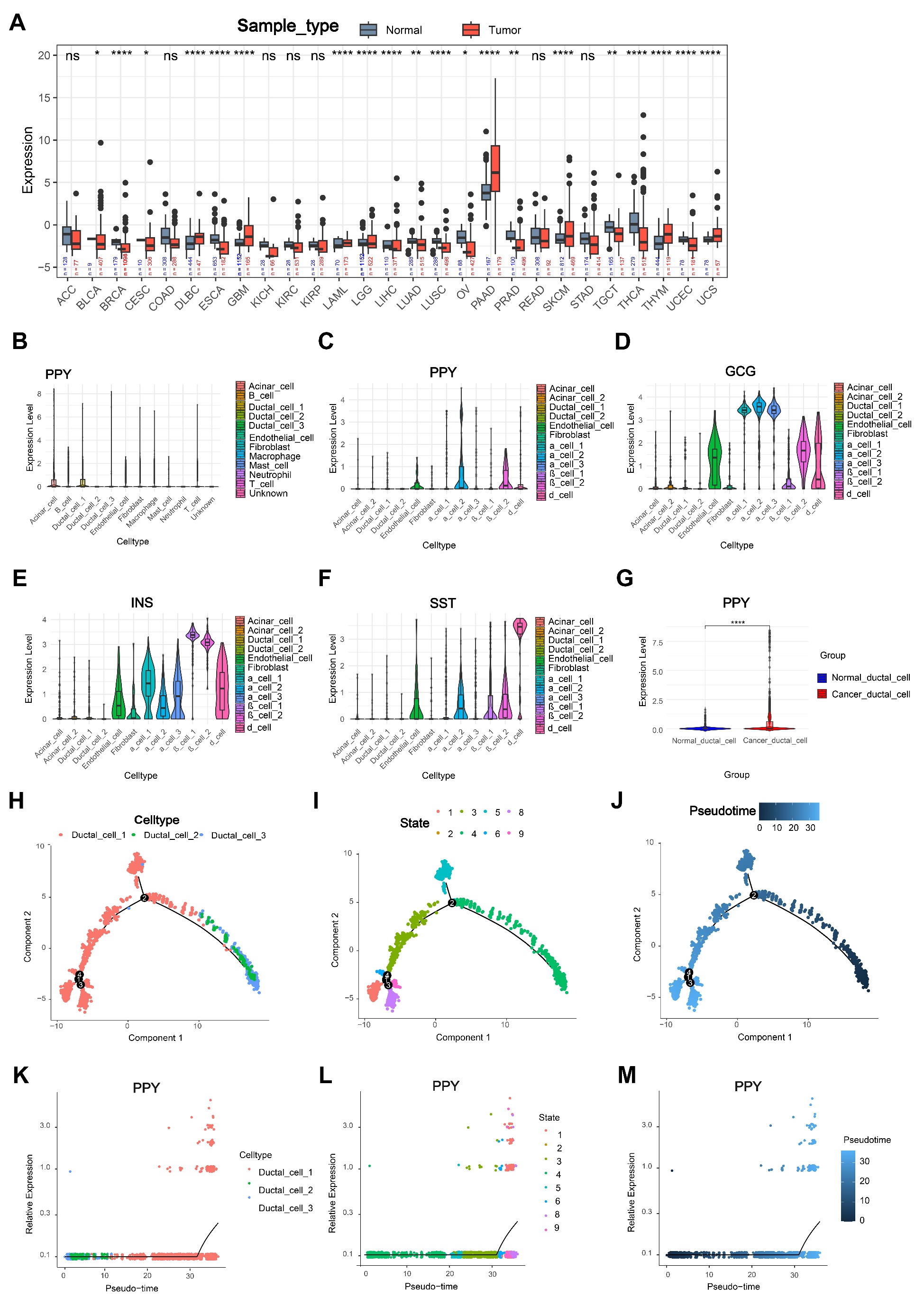


**Supplementary Figure S5. The expression characteristics of PPY in normal and cancer tissues. A ,** The mRNA level of PPY in global human cancer tissues (red) and non-tumor tissues (blue) were analyzed using samples from the TCGA database and the GTEx database. **B,** Violin plot illustrating the expression levels of PPY across distinct cell populations in PDAC sc-RNA data. **C-F,** Violin plot illustrating the expression levels of PPY (C), GCG (D), and INS (E), SST (F) across distinct cell populations in normal pancreas sc-RNA data. **G,** The expression levels of PPY in the ductal cells of PDAC and normal pancreas were extracted from their respective scRNA-seq datasets, and a violin plot was utilized to illustrate the differences in PPY expression. **H-J,** A pseudotime trajectory plot generated by Monocle2, illustrating the developmental progression of 3 groups of ductal cells in PDAC sc-RNA data, and the Cell type (H), State (I), and Pseudotime (J) are colored separately on the trajectories. **K-M,** Line plots showing the expression profiles of PPY along the pseudotime trajectory, and the Cell type (K), State (L), and Pseudotime (M) are colored separately on the trajectories. The results are presented as mean ± SD; *, P < 0.05; **, P < 0.01; ***, P < 0.001; ***, P < 0.001; ns, not statistically significant.


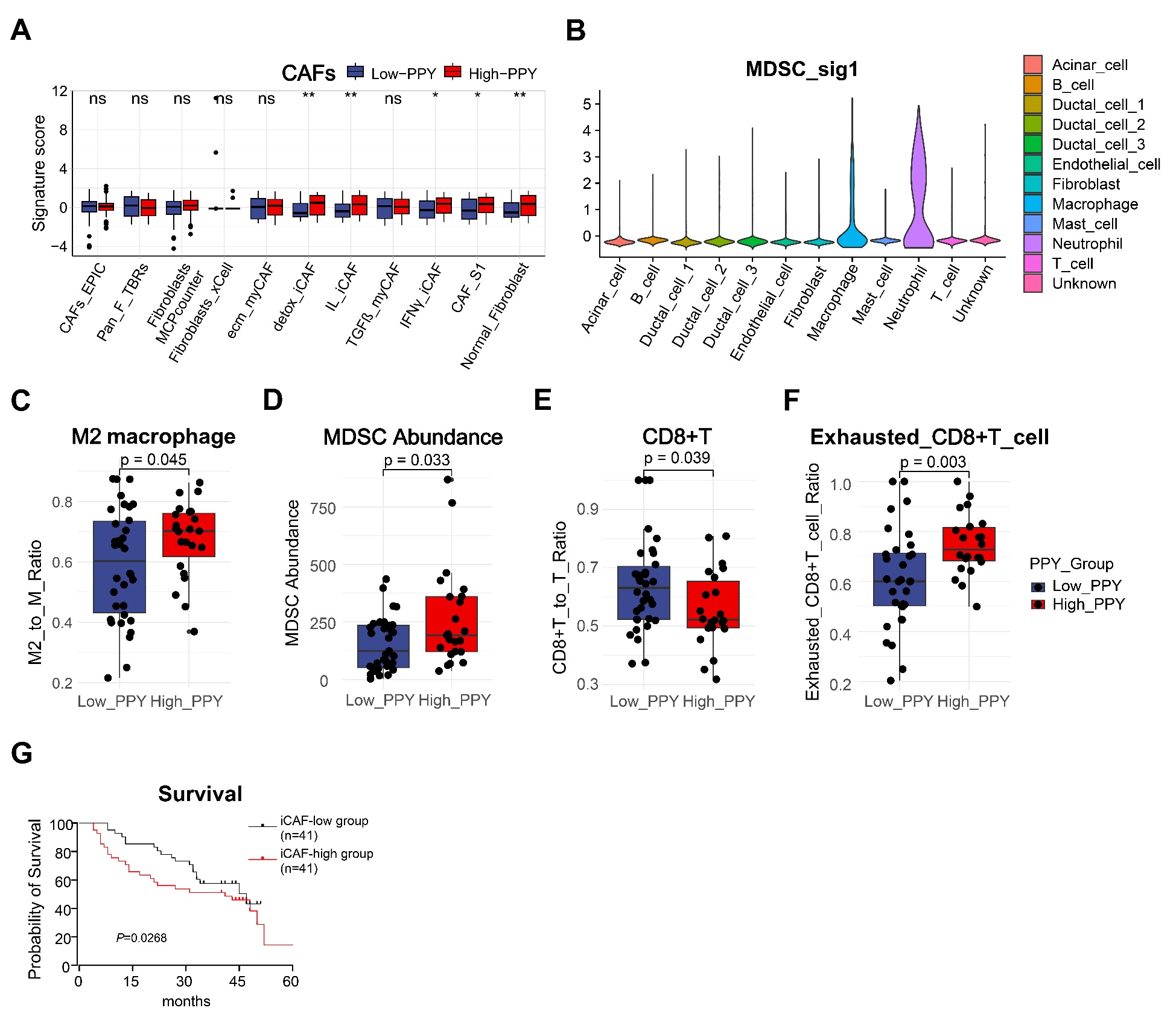


**Supplementary Figure S6. The correlation of PPY expression with the TME of PDAC, and the correlation of iCAF abundance with prognosis. A,** The correlation between PPY expression and TME signature was analyzed using IOBR tool. **B,** Violin plots illustrate the distribution of MDSC feature scores across various cell populations within the PDAC sc-RNA seq data. **C-F,** The abundance of M2 macrophage (C), MDSC abundance (D), and CD8+ T cells (E) and exhausted CD8+T proportion (F) in the TME of PPY-low and PPY-high group were analyzed using PDAC sc-RNA seq data. **G,** The correlation between iCAFs and overall survival time was analyzed utilizing multiplex immunohistochemistry (mIHC) data. The results are presented as mean ± SD; *, P < 0.05; **, P < 0.01; ***, P < 0.001; ***, P < 0.001; ns, not statistically significant.

**
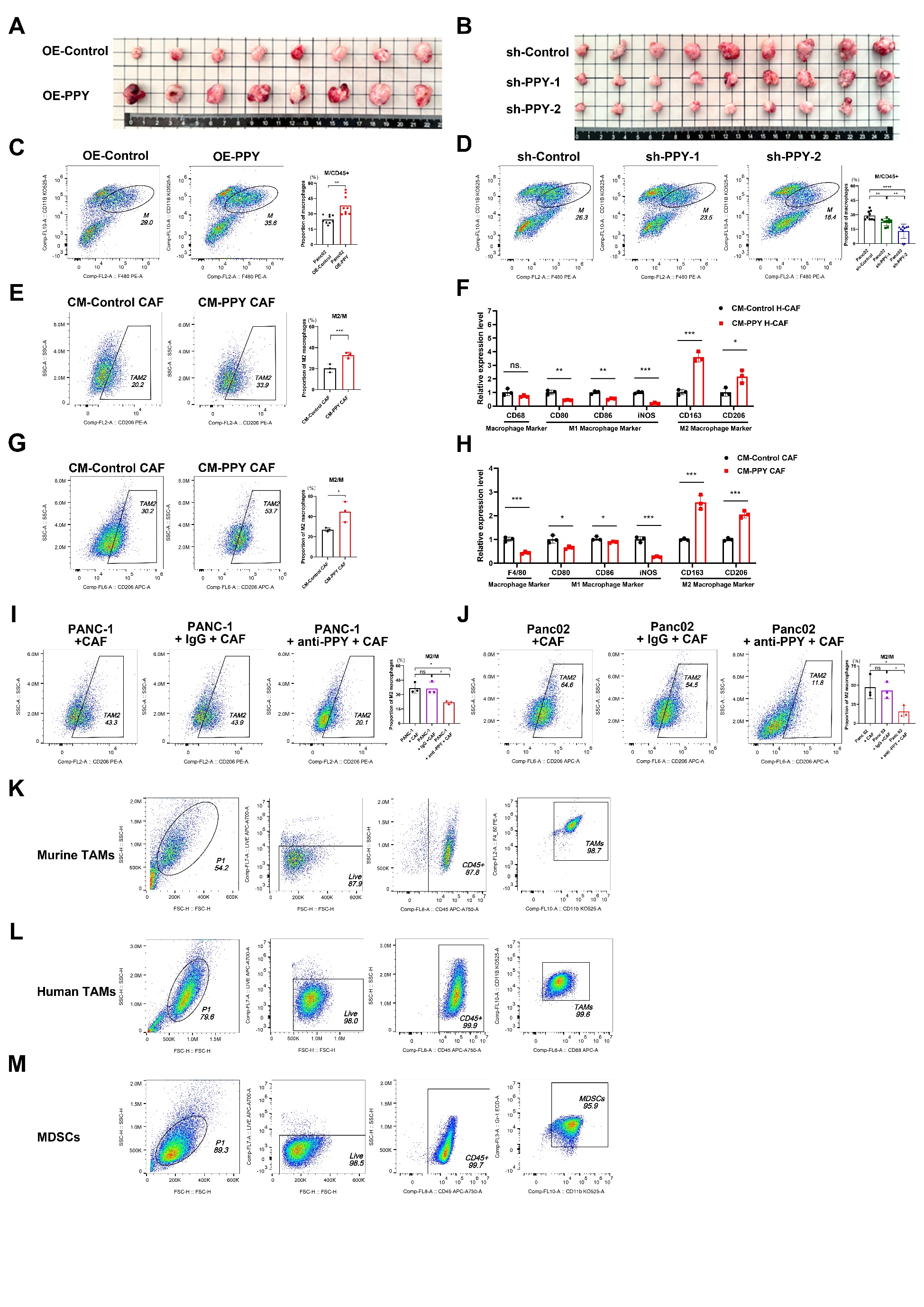
**

**Supplementary Figure S7. PPY-induced iCAFs reprogram the immune microenvironment of PDAC and promote cancer progression.** **A,** The gross image of the orthotopic allograft tumor model that was established by conjecting KPC mouse-derived CAFs and cancer cells with upregulated expression of PPY or respective control cells. **B,** The gross image of the orthotopic allograft tumor model that was established by conjecting KPC mouse-derived CAFs and cancer cells with down-regulated expression of PPY or respective control cells. **C and D,** Flow cytometry analysis of the infiltration of macrophages in the TME of the PPY upregulation group (C), down-regulation group (D), and their respective control group. **E-H,** After co-culturing human macrophages (E and F) and mouse macrophages (G and H) with CAFs that were respectively treated with PPY supernatant, flow cytometry (E and G) and qRT-PCR (F and H) were applied to analyze the M2 macrophage polarization in these macrophages. **I** and **J,** Human CAFs and mouse CAFs were separately co-cultured with cancer cells in the presence of PPY antibody, followed by co-culture with human macrophages (I) and mouse macrophages (J). Flow cytometry was applied to analyze the M2 macrophage polarization in these macrophages. **K and L**, Flow cytometry analysis of purity of murine macrophages (K), human macrophages (L), and MDSCs (M). The statistical data is presented as mean ± SD and analyzed using the unpaired t-test. *, *P* < 0.05; **, *P* < 0.01; ***, *P* < 0.001; ***, *P* < 0.001; ns, not statistically significant.


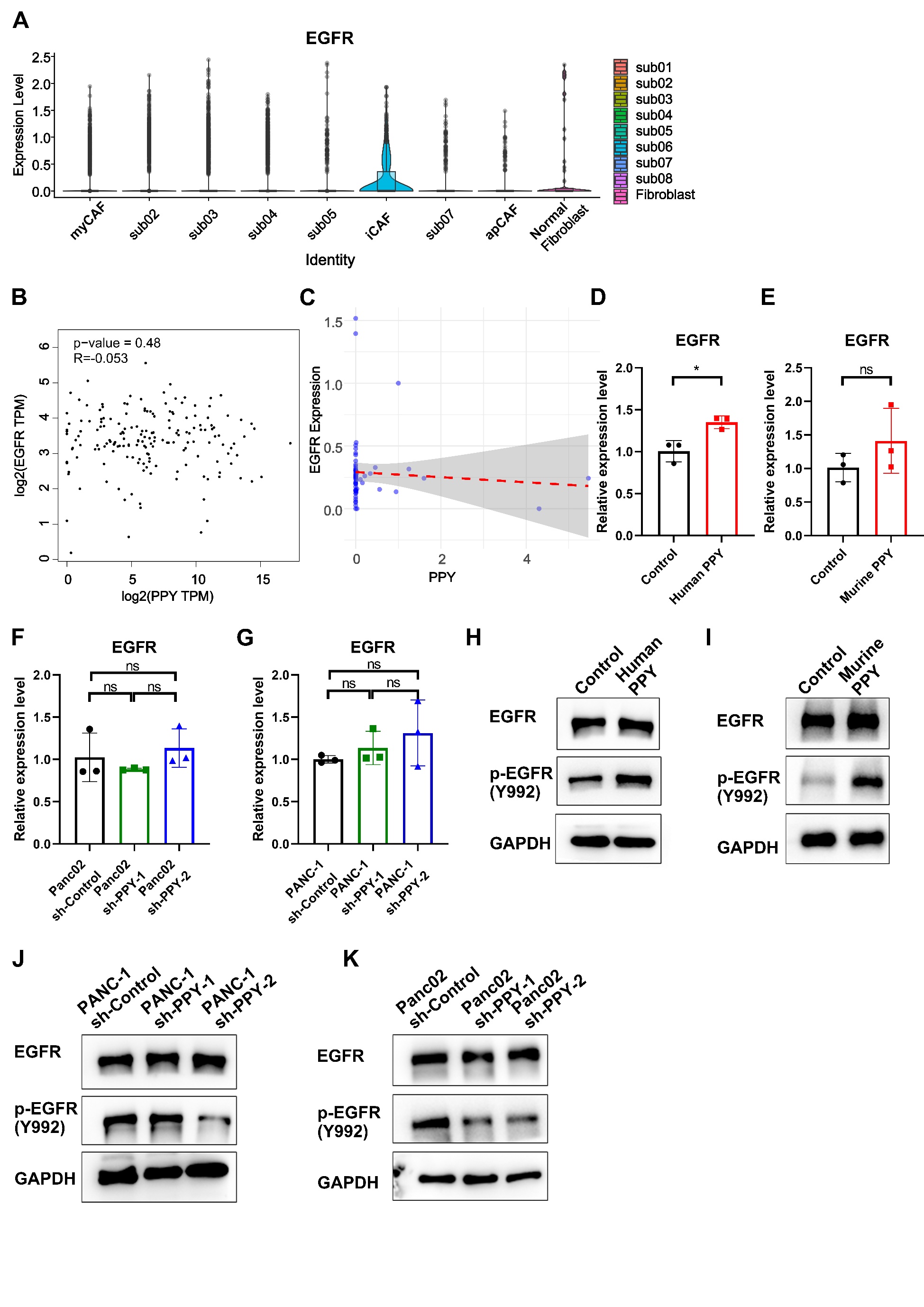


**Supplementary Figure S8. EGFR expression in distinct CAF subsets and the correlation of EGFR expression and activation with PPY expression. A,** Violin plots illustrate the EGFR expression across different CAF subsets within the PDAC sc-RNA seq data. **B and C,** The mRNA expression correlation between EGFR and PPY was analyzed using PDAC bulk RNA-seq data from GEPIA (B) and PDAC scRNA-seq data from GEO (C). **D and E,** qRT-PCR analyses of EGFR expression in human (D) and murine CAFs (E) after treating them with recombinant PPY proteins respectively. **F and G,** qRT-PCR analyses of EGFR expression in human CAFs (F) and murine CAFs (G) following co-culture with cancer cells that had downregulated PPY expression. **H and I,** Immunoblotting analyses of EGFR expression in human (H) and murine CAFs (L) after treating them with recombinant PPY proteins respectively. **J and K,** Immunoblotting analyses of EGFR expression in human (J) and murine CAFs (K) following co-culture with cancer cells that had downregulated PPY expression.
